# Genetic dissection of protein and starch during wheat grain development using QTL mapping and GWAS

**DOI:** 10.3389/fpls.2023.1189887

**Published:** 2023-06-12

**Authors:** Yingxin Guo, Guanying Wang, Xin Guo, Songqi Chi, Hui Yu, Kaituo Jin, Heting Huang, Dehua Wang, Chongning Wu, Jichun Tian, Jiansheng Chen, Yinguang Bao, Weidong Zhang, Zhiying Deng

**Affiliations:** ^1^ State Key Laboratory of Wheat Breeding, Group of Wheat Quality and Molecular Breeding, College of Agronomy, Shandong Agricultural University, Tai’an, Shandong, China; ^2^ College of Biological and Chemical Engineering, Qilu Institute of Technology, Jinan, Shandong, China; ^3^ Taiyuan Agro-Tech Extension and Service Center, Taiyuan, Shanxi, China; ^4^ R&D Department, Shandong Huatian Agricultural Technology Co., Ltd, Feicheng, Shandong, China

**Keywords:** wheat, protein, starch, GMP, QTL mapping, GWAS

## Abstract

Protein, starch, and their components are important for wheat grain yield and end-products, which are affected by wheat grain development. Therefore, QTL mapping and a genome-wide association study (GWAS) of grain protein content (GPC), glutenin macropolymer content (GMP), amylopectin content (GApC), and amylose content (GAsC) were performed on wheat grain development at 7, 14, 21, and 28 days after anthesis (DAA) in two environments using a recombinant inbred line (RIL) population of 256 stable lines and a panel of 205 wheat accessions. A total of 29 unconditional QTLs, 13 conditional QTLs, 99 unconditional marker−trait associations (MTAs), and 14 conditional MTAs significantly associated (*p* < 10^−4^) with four quality traits were found to be distributed on 15 chromosomes, with the phenotypic variation explained (PVE) ranging from 5.35% to 39.86%. Among these genomic variations, three major QTLs [QGPC3B, QGPC2A, and QGPC(S3|S2)3B] and SNP clusters on the 3A and 6B chromosomes were detected for GPC, and the SNP TA005876-0602 was stably expressed during the three periods in the natural population. The QGMP3B locus was detected five times in three developmental stages in two environments with 5.89%–33.62% PVE, and SNP clusters for GMP content were found on the 3A and 3B chromosomes. For GApC, the QGApC3B.1 locus had the highest PVE of 25.69%, and SNP clusters were found on chromosomes 4A, 4B, 5B, 6B, and 7B. Four major QTLs of GAsC were detected at 21 and 28 DAA. Most interestingly, both QTL mapping and GWAS analysis indicated that four chromosomes (3B, 4A, 6B, and 7A) were mainly involved in the development of protein, GMP, amylopectin, and amylose synthesis. Of these, the wPt-5870–wPt-3620 marker interval on chromosome 3B seemed to be most important because it played an important role in the synthesis of GMP and amylopectin before 7 DAA, in the synthesis of protein and GMP from 14 to 21 DAA, and in the development of GApC and GAsC from 21 to 28 DAA. Using the annotation information of IWGSC Chinese Spring RefSeq v1.1 genome assembly, we predicted 28 and 69 candidate genes for major loci from QTL mapping and GWAS, respectively. Most of them have multiple effects on protein and starch synthesis during grain development. These results provide new insights and information for the potential regulatory network between grain protein and starch synthesis.

## 1 Introduction

Wheat (*Triticum aestivum* L.) is one of the most important cereal crops in the world. It is the main source of carbohydrates, protein, and dietary fiber for humans (Ma et al., 2013). Protein and starch are important components of wheat grain and affect both the yield and processing quality of wheat. Wheat grains contain 8%–20% protein and 85% carbohydrates, most of which are starch (Anjum et al., 2007). In recent years, with the continuous improvement of human dietary habits and living standards, more attention has been given to the nutritional composition and flavor quality of wheat products. In addition, increasingly extreme weather conditions and an increasing world population impose a severe threat to wheat production (Zhang et al., 2021). Therefore, on the basis of ensuring high yield, breeding new wheat varieties with high quality and wide adaptability is the main goal of wheat quality breeding worldwide (Liu et al., 2018). It is of great significance to study and analyze the basic mechanism or important genes/loci affecting protein and starch content in wheat grain development.

The grain protein content (GPC) is important for dough and end-use quality because it influences the ratio of gliadin to glutenin. Therefore, improving GPC has become an important goal of wheat quality breeding, but previous studies showed that it was affected not only by genetics but also by the environment (Paull and Anderson, 1942). GPC is regarded as a quantitative trait. Several studies have reported quantitative trait loci (QTL) for GPC and flour protein content using different populations, which involved approximately 19 chromosomes. The majority of these QTLs were reported on chromosomes 6B and 7B. Of these, the marker *Gpc-B1* on chromosome 6B has been used in wheat quality breeding. Two important QTLs, *QGpc.ink-2B* and *QGpc.ink-6A*, were detected for GPC by GWAS. The *QGpc.ink-6A* locus controlled both GPC and GSC (grain starch content) with opposite allelic effects (Muqaddasi et al., 2020). However, little is known about the genes determining the inheritance of GPC in wheat; the only identified causative gene in a GPC locus associated with high GPC yield thus far is an NAC transcription factor that originated from wild emmer wheat (Souer et al., 1996; Aida et al., 1997). In wheat, the endosperm-specific transcription factor TaNAC019 was found to affect grain quality by regulating glutenin and starch accumulation (Gao et al., 2021). Therefore, the identification of genetic loci or genes controlling GPC is helpful for the effective breeding of wheat varieties with high protein synthesis and lays a foundation for an in-depth understanding of the regulation between wheat protein and starch synthesis.

Polymeric glutenins are an important component of gluten proteins in wheat synthesized in starchy endosperm cells during grain filling. They contribute to the elasticity of gluten. The disulfide bonds of glutenin subunits are present in the glutenin macropolymer (GMP), allowing it to play an important role in the establishment of the gluten network structure when it achieves a certain size (Tao et al., 2022). It mainly comprises high- molecular-weight glutenin subunits (HMW-GS) and low- molecular-weight glutenin subunits (LMW-GS). The effects of the GMP, HMW-GS, and LMW-GS on dough and processing quality have been extensively studied, and most of them greatly contribute to wheat quality (Ma et al., 2005). Although genetic factors, such as the ratio of HMW-GS to LMW-GS and their allelic variations, affect the GMP and thus the viscoelastic properties of gluten, the GMP is also affected by the interaction between genotype and environment. The GMP is a quantitative trait. Few studies have been reported on the genes/loci for the GMP by QTL mapping or GWAS. Two major QTL clusters for glutenin have been reported, *1DL-2* and *6AS-3*, at the chromosome regions 409.26–416.45 Mb and 44.02–50.35 Mb, respectively. HMW-GS and LMW-GS were detected, and Kompetitive Allele-Specific PCR (KASP) markers were developed using a recombinant inbred line population (Zhou et al., 2021). However, little is known about how many genes are involved in wheat grain development and how they work during that process.

Starch is a major storage component of wheat grain endosperm. Common wheat has an amylose content of 25%–30% and an amylopectin content of 70%–75%, both of which are very important for processing and end-use quality. Some studies showed that the GSC was positively correlated with the increase in grain size and eventually grain yield but was negatively correlated with the GPC, which indicated that a balance must be achieved to obtain the desired protein level and high yield (Muqaddasi et al., 2020). In addition, many key enzymes of starch synthesis, such as granule-bound starch synthase I (GBSS I), soluble starch synthase (SSS), branching enzyme (BE), and debranching enzyme (DBE), were found in wheat. Although some key enzymes were found, the genes determining the inheritance of GSC in bread wheat are still not clear. In addition to the known *waxy* genes, many other genes/QTLs should be further investigated. A few studies on QTL mapping of GSC have been reported, and the QTLs were mainly distributed on chromosomes 1A, 1D, 2A, 2D, 4A, 7A, 7B, and 7D (Tian et al., 2015). A major QTL for the B-type granule content was detected in wild Aegilops species (Muqaddasi et al., 2020). In addition, a genome-wide association study (GWAS) using the 90 K SNP assay identified 23 loci for percentage volumes of A- and B-granules and 25 loci for the ratio of A-/B-granule volumes, distributing on 15 chromosomes (Li et al., 2017). These findings support the hypothesis that wheat may alter grain starch content by inducing or inhibiting different genes.

The development of wheat grain is a complex process involving cellularization, grain filling, and maturation. Grain filling is a major stage for the synthesis and accumulation of storage molecules, such as starch and gluten proteins (Nadaud et al., 2010). Different gene expression patterns exist for the seed coat, endosperm, and embryo during the grain filling process (Brandizzi, 2021). The discovery of the conditional QTL mapping method laid a foundation for analyzing the dynamics of QTL/gene expression (Zhu, 1995; Bethke et al., 1996). The accumulations of protein and starch showed dynamic and orderly changes over a considerable time during the development of wheat grain. Therefore, previous researchers used this method to study the genetic basis of crops, such as cotton, rice, maize, soybeans, and wheat (Jiang et al., 2008; Wang et al., 2010; Cui et al., 2011; Wang et al., 2012; Zhang et al., 2017). In a few studies, conditional QTLs of GSC and GPC during grain development in DH populations were mapped. *QGpc3A*, *QGpc4A-1*, *QGpc1D*, and *QGpc4A-2* were found to play the most important role in the accumulation of GPC, and *QGsc4A* was found to play an important role in the accumulation of starch (Tian et al., 2015). Two conditional QTLs related to protein content were detected at the grain filling stage. This is a good start for the study of the regulation of protein and starch content during wheat grain development by conditional QTLs (Li et al., 2015).

In addition to QTL mapping, genome-wide association studies (GWAS) based on high-density molecular markers are a powerful tool for determining the genetic anatomy of complex traits. They are an effective method to study the economic and biological values of germplasm resources (Alqudah et al., 2020). GWAS depend on the association between molecular markers and the phenotype of the target trait. The combined analysis of QTL mapping and GWAS can improve the accuracy of QTL/gene detection and has been used in rice (Famoso et al., 2011; Zhai et al., 2018; Pan et al., 2020; Chen et al., 2022) and maize (Wang et al., 2019; Li Z. et al., 2020). However, in wheat, few studies using this combination analysis have been reported (Chen et al., 2016), and the following genes were detected: *TaFLL-5B1* for flag leaf length (Yan et al., 2020), *qHR-1B* for herbicide resistance (Shi et al., 2020), and *S2B_26494801* for drought tolerance of winter wheat (Sallam et al., 2022). These results indicate that this combination analysis could be feasible for identifying QTLs/genes for protein, starch, and their components during wheat grain development.

Although genetic mapping of protein content, starch content, and their components in wheat grain has been performed, most studies used mature grain as the study material, not developing grains. Moreover, there are almost no reports on determining the development of grain protein, GMP, GAPC, and GASC using conditional and unconditional mapping by the combined analysis of QTL mapping and GWAS. Therefore, the objective of this study was to identify the important dynamic QTL/gene changes of wheat grain traits at the molecular level during continuous development under two environments at 7, 14, 21, and 28 DAA (days after anthesis) by combining conditional and unconditional QTL mapping and GWAS analysis. The results help explain the developmental genetic mechanism for regulating the contents of protein, starch, and their components and the GMP, which provides new insights into the potential regulatory network between grain quality and yield.

## 2 Materials and methods

### 2.1 Plant materials

The data presented in this study were derived from one recombinant inbred line (RIL) population and an association mapping panel comprising 205 wheat genotypes.

The RIL population of 256 lines was developed from a cross between the two winter wheat cultivars Nuomai 1 (female) (NM1) and Gaocheng 8901 (male) (GC8901) by the single seed descent method (Deng et al., 2015). NM1 (Jiangsu Baihuomai/Guandong107) has unique starch properties that are related to high-quality white salt noodles. GC8901 (77546-2/Lin zhang) exhibits high gluten strength and good bread-making qualities. The association mapping panel comprised 77 released cultivars, 55 landraces, and 73 breeding lines. Most of them are from 10 provinces that represent the major winter wheat production regions in China (Chen et al., 2016).

### 2.2 Growth conditions

The seeds of the RIL population and association mapping panel were planted in the experimental fields at Shandong Agricultural University, Tai’an location (36°57′N, 116°36′E) for two growing seasons, 2016–2017 and 2017–2018. The experiments were laid in a completely randomized block design with two replicates. Under each environment, the RILs, parents, and natural wheat lines were grown in 2-m-long four-row plots spaced 26 cm apart. During the growing season, field management was in accordance with local practices, and the plants were not damaged by disease or insects.

The four sampling stages are indicated by S1, S2, S3, and S4. S1 indicates 7 DAA, S2 indicates 14 DAA, S3 indicates 21 DAA, and S4 indicates 28 DAA.

### 2.3 Mill flour

The wheat spikes were tagged at the flowering stage and harvested at 7, 14, 21, and 28 DAA. Among them, 32 spikes were harvested at 7 DAA, 24 spikes at 14 DAA, 18 spikes at 21 DAA, and 28 spikes at 28 DAA. The harvested wheat spikes were faded at 105°C for 10 min and then dried at 80°C before threshing. The wheat grains were ground into whole flour by an LM3100 experimental mill and then stored at 4°C for use.

### 2.4 Quality trait measurements

Whole flour protein content (FPR) was measured by the Kjeldahl method according to GB/T 33862-2017.

Flour starch content of wheat samples was measured by the double-wave method (Chen et al., 2019).

The method from Sun’s study was used to determine the GMP content in grains with minor modifications (Sun et al., 1998). Whole wheat flour (0.1 g) was placed into a 10-ml centrifuge tube, and 5 ml of 1.5% SDS extract was added. The mixture was shaken for 2 h and centrifuged at 15,500 r/min at room temperature for 15 min. The supernatant was discarded, and the residual nitrogen content was determined by the biuret method. Four milliliters of biuret reagent diluted once and 1 ml of distilled water were added to the mixture, and it was shaken for 2 h and then centrifuged. The supernatant was used to measure the light absorption value at 540 nm, and the nitrogen content in the residue was used as the approximation of GMP.

### 2.5 The genetic linkage map

The linkage map of the RIL population was constructed in a previous study (Deng et al., 2016). A total of 501 markers were mapped, including 479 DArT markers, 17 SSR markers, 2 HMW-GS markers, and 3 Wx protein markers. It included 21 chromosomes comprising 25 linkage maps and covered 4,213.2 cM with an average marker distance of 8.4 cM.

SNP markers, genotyping, and the population structure of the association mapping panel have been previously reported with the high-density illumina wheat 90K SNP array (Chen et al., 2016). In total, 24,355 mapped SNP markers were used for the MTA analysis.

### 2.6 Statistical analyses

PASW Statistics 18 software was used for statistical analysis of population traits, QTL IciMapping 3.2 software (https://isbreeding.caas.cn/rj/qtllcmapping/294441.htm, released April 2012) was used for linkage analysis of RIL wheat lines, and a mixed linear model (MLM) in TASSEL3.0 software (Zhang et al., 2010) was used for identifying significant marker−trait associations (MTAs). The *p*-value was used to determine whether an MTA locus was associated with a marker. The *R* ^2^ value was used to evaluate the magnitude of the MTA effects. The genome-wide significance threshold (*p* < 0.0001) was determined. When the MTA locus was detected in two or more environments, it was considered a stable association site. QGAStation 1.0 (http://ibi.zju.edu.cn/software/qga/) was used to calculate the conditional phenotypic values, which were subsequently used as input data for conditional QTL mapping.

To clarify the designations of the examined QTLs, the following rules were adopted: “Q” denotes “QTL”; the letter following “Q” is an abbreviation of its corresponding trait; a number followed by an upper case letter, “A,” “B,” or “D,” represents the chromosome in a given wheat genome where the corresponding QTL was detected; if there is more than one QTL on one chromosome, a serial number behind a hyphen is added (e.g., *QGApC3B-1* stands for the second QTL for grain amylopectin content detected on chromosome 3B); and the growth stages are added before the chromosome number for conditional QTLs [e.g., *QGApC (S4|S3)3B*].

### 2.7 Forecasting candidate genes for major QTL/MTA loci during grain development

To identify the position of important QTLs on a physical map and possible candidate genes, significant markers detected in this study were used to identify putative candidate genes according to the method of Ji et al. (2021). A BLAST (Basic Local Alignment Search Tool) search was performed on the WheatOmics1.0 database (Ma et al., 2021) by Chinese Spring IWGSC RefSeq v1.1 genome assembly (http://wheatomics.sdau.edu.cn/blast/blast.html) using the sequence of the significant DArT markers and SNP markers identified by QTL mapping and GWAS. When a DArT marker or SNP marker sequence from the IWGSC was 100% identical to any wheat contig, the sequence was extended 2 Mb using the IWGSC BLAST results (Ji et al., 2021). Then, the extended sequence was used to run a BLAST search of the National Center for Biotechnology Information (NCBI) database (http://www.ncbi.nlm.nih.gov) and Ensembl Plants (http://plants.ensembl.org/Triticum_aestivum/Tools/Blast) to confirm possible candidate genes and functions.

## 3 Results

### 3.1 Phenotypic data and correlations

During grain development, the dynamic changes of GPC and GMP content in both the RIL population and the diversity panel showed basically consistent trends of “high–low–high” in the two environments (**Tables S1**, **S2**). However, the dynamic changes in amylopectin content (GApC) and amylose content (GAsC) in the RIL population and natural population were greatly affected by the environment and showed a gradual upwards trend (**Tables S1**, **S2**). All of the evaluated traits in the RIL population exhibited approximately continuous variation in different developmental stages in the two environments (**Figures S1–S4**), and transgressive segregation was observed for all the evaluated traits in these populations, indicating that alleles with positive effects were contributed by both parents.

By correlation analysis, a significantly positive correlation was observed between GPC and GMP content, but GPC was significantly negatively correlated with GApC and GAsC (**Table S3**). There was a significantly negative correlation between GMP content and GApC, but a significant positive correlation was found between GMP content and GAsC.

### 3.2 QTL mapping of four evaluated traits during grain development

A total of five unconditional QTLs for GPC were identified on chromosomes 1B, 2A, 3B, 3D, and 7A (**Table 1**). There were two major QTLs, *QGPC3B* and *QGPC2A*, with flanking markers of *wPt-5870–wPt-3620* and *wPt-2994–wPt-0071*, accounting for 26.97% and 12.76% of the phenotypic variation explained (PVE), respectively. They were detected at 21 DAA and 28 DAA, respectively. The additive effects of the two loci were from the male parent Gaocheng 8901.

**Table 1:** Unconditional QTL analysis of the content of protein, GMP, amylopectin, and amylose during grain development in the RIL population.

Env.	Trait	Period	QTL	Position	Marker interval	LOD	PVE (%)	Add
E1	GPC	S3	*QGPC3B*	164	*wPt-5870 –wPt-3620*	6.91	26.97	−2.21
	GMP	S1	*QGMP3B*	164	*wPt-5870 –wPt-3620*	9.81	33.62	−13.52
		S3	*QGMP3B*	164	*wPt-5870 –wPt-3620*	4.53	17.77	3.44
		S3	*QGMP7B*	0	*wPt-665293 –wPt-4038*	3.07	5.82	−0.56
		S4	*QGMP2A*	219	*wPt-3896 –wPt-2644*	2.77	12.40	3.85
	GApC	S1	*QGApC4A*	156	*wPt-6404 –wPt-2291*	3.03	6.33	−3.81
		S4	*QGApC7D*	0	*Wx-D1 –wPt-664368*	2.73	5.82	−2.16
	GAsC	S2	*QGAsC7A*	162	*wPt-731311 –Wx-A1*	2.52	5.49	−0.36
		S4	*QGAsC1B*	170	*wPt-6442 –wPt-3824*	2.60	5.35	0.33
		S4	*QGAsC4A*	103	*Wx-B1 –wPt-0105*	2.97	6.03	−0.35
E2	GPC	S2	*QGPC7A*	159	*wPt-731311 –Wx-A1*	3.50	8.92	0.51
		S3	*QGPC1B*	0	*wPt-3563 –wPt-8226*	3.18	7.24	0.29
		S4	*QGPC2A*	268	*wPt-2994 –wPt-0071*	2.62	12.76	−0.43
		S4	*QGPC3D*	50	*xgpw-4136 –xgwm-3*	2.61	8.10	−0.30
	GMP	S1	*QGMP6A*	69	*wPt-1642 –wPt-4229*	2.54	6.24	1.02
		S2	*QGMP1D*	16	*Glu-D1 –wPt666719*	3.08	5.94	−0.75
		S2	*QGMP3B*	164	*wPt-5870 –wPt-3620*	3.12	5.89	2.12
		S3	*QGMP3B*	164	*wPt-5870 –wPt-3620*	2.77	8.66	2.37
		S3	*QGMP3D*	48	*xgpw-4136 –xgwm-3*	5.23	17.95	−1.05
		S4	*QGMP4A*	75	*wPt-4230 –wPt-731137*	2.68	6.10	−0.54
	GApC	S1	*QGApC3B.1*	191	*wPt-5072 –wPt-9752*	3.90	25.69	−6.13
		S2	*QGApC2A*	251	*wPt-9951 –wPt-2273*	2.93	8.15	4.57
		S3	*QGApC4A*	102	*Wx-B1 –wPt-0105*	3.23	7.35	−2.61
		S3	*QGApC7D*	1	*Wx-D1 –wPt-664368*	3.82	8.97	−2.88
		S4	*QGApC3B.2*	319	*wPt-8206 –wPt-9368*	2.84	5.88	2.80
	GAsC	S3	*QGAsC1D*	112	*wPt-665814 –wPt-3738*	3.12	15.55	0.51
		S3	*QGAsC3B*	164	*wPt-5870 –wPt-3620*	4.63	10.98	−0.99
		S3	*QGAsC7D*	0	*Wx-D1 –wPt-664368*	3.42	6.57	−0.33
		S4	*QGAsC3B*	164	*wPt-5870 –wPt-3620*	6.73	18.36	−1.87

E1 means 2016–2017, E2 means 2017–2018, S1 means 7 days after flowering, S2 means 14 days after flowering, S3 means 21 days after flowering, and S4 means 28 days after flowering.

There were 12 QTLs found for GMP content on chromosomes 1D, 2A, 3B, 4A, 6A, and 7B (**Table 1**). The major QTL, *QGMP3B*, was stably detected at three stages over 2 years, which indicated that this QTL was important for the development of GMP. The position of this locus was the same as that of *QGPC3B*, which indicated that this locus is important for protein synthesis.

Seven QTLs were identified for GApC on chromosomes 2A, 3B, 4A, and 7D (**Table 1**). Of these, *QGApC7D* was found with flanking markers between *Wx-D1* and *wPt-664368* at 28 DAA and 21 DAA in E1 and E2, respectively. Although there were two QTLs identified on chromosome 4A, they were not mapped to the same marker intervals. *QGApC4A.2* was detected between markers *Wx-B1* and *wPt-0105* at 21 DAA in E2. On chromosome 3B, one major QTL, *QGApC3B.1*, was identified with 25.69% PVE.

**Figure 1:**
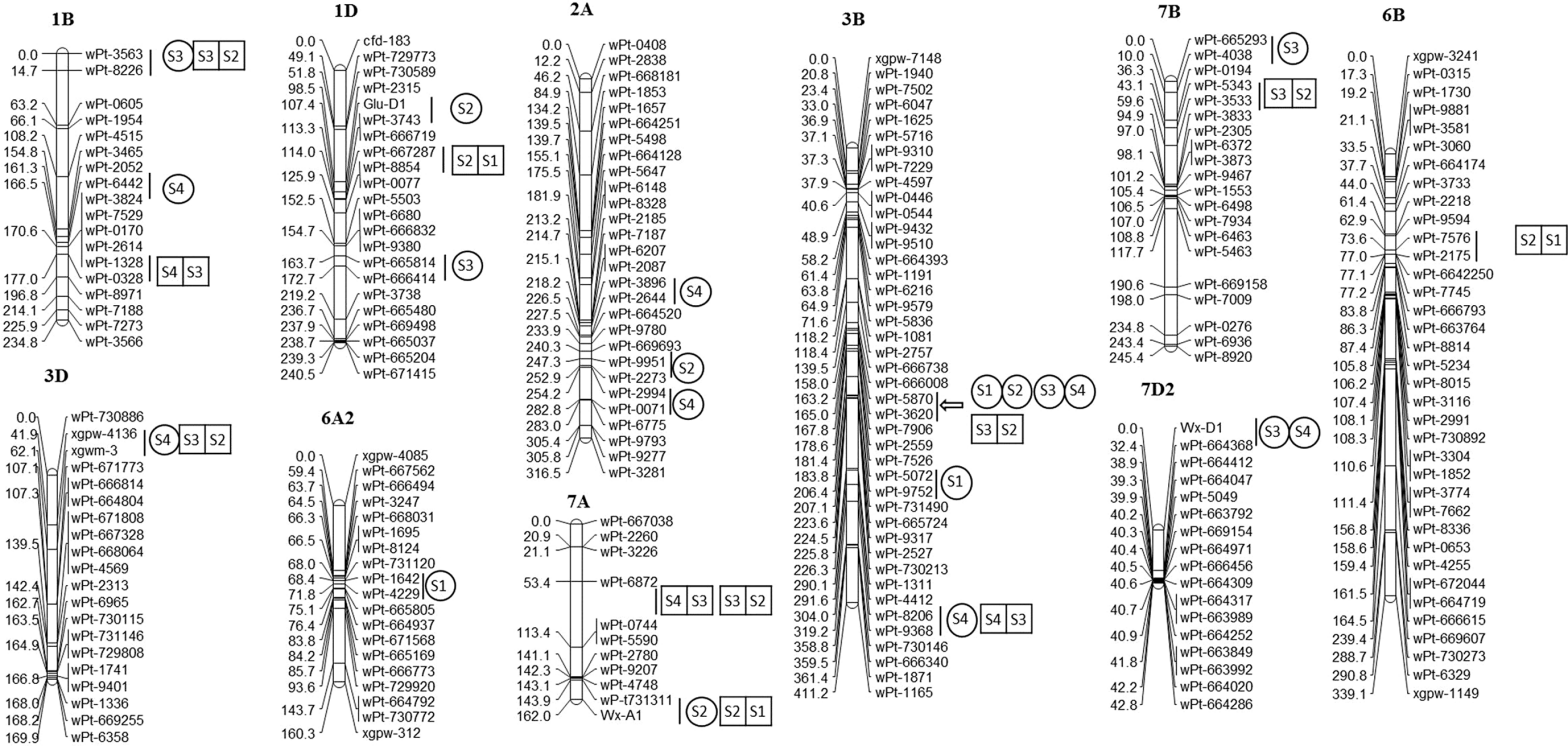
QTLs for four quality traits in the RIL population at four stages in 2 years. 
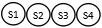
represent unconditional QTLs at four periods, respectively; 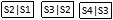
represent conditional QTLs at four periods, respectively.

A total of eight unconditional additive QTLs for GAsC were detected on chromosomes 1B, 1D, 3B, 4A, 7A, and 7D (**Table 1**; **Figure 1**). A major QTL, *QGAsC3B*, was found at 21 DAA and 28 DAA in the E2 environment, with flanking markers between *wPt-5870* and *wPt-3620*, which indicated that this QTL was important for GAsC.

A total of 13 conditional QTLs were identified in RIL wheat lines at four periods in 2 years (**Table 2**). Four QTLs for GPC were detected on chromosomes 1B, 3B, 7A, and 7B in 2 years. There were two major QTLs, *QGPC(S3|S2)3B* and *QGPC(S3|S2)7A*, with 29.17% and 14.42% PVE, respectively. Four conditional additive QTLs for GMP content were found on chromosomes 3B, 3D, and 7A, including three major QTLs, *QGMP(S3|S2)3B*, *QGMP(S3|S2)3D*, and *QGMP(S4|S3)7A*. For GApC, only one major QTL, *QGApC (S4|S3)3B*, was identified in E2, with 14.30% PVE. Four conditional additive QTLs for GAsC were found on chromosomes 1B, 1D, 6B, and 7A. For GAsC, there was one major QTL, *QGAsC(S4|S3)7A*, with a PVE of 21.86%.

**Table 2:** Conditional QTL analysis of the content of protein, GMP, amylopectin, and amylose during grain development in the RIL population.

Env	Trait	Condition treatment	QTL	Position	Marker interval	LOD	PVE (%)	Add
E1	GPC	S3|S2	*QGPC(S3|S2)3B*	162	*wPt-5870 –wPt-3620*	5.72	29.17	−3.99
	GMP	S3|S2	*QGMP(S3|S2)3B*	164	*wPt-5870 –wPt-3620*	4.50	18.38	3.64
	GAsC	S2|S1	*QGAsC(S2|S1)1D*	28	*wPt-667287 –wPt-8854*	2.55	7.65	0.41
		S2|S1	*QGAsC(S2|S1)6B*	76	*wPt-7576 –wPt-2175*	2.93	8.81	−0.45
		S4|S3	*QGAsC(S4|S3)1B*	170	*wPt-6442 –wPt-3824*	3.07	8.47	0.37
		S4|S3	*QGAsC(S4|S3)7A*	100	*wPt-6872 –wPt-0744*	3.32	21.86	−0.60
E2	GPC	S3|S2	*QGPC(S3|S2)1B*	0	*wPt-3563 –wPt-8226*	3.26	8.28	0.30
		S3|S2	*QGPC(S3|S2)7A*	59	*wPt-6872 –wPt-0744*	3.64	14.42	0.39
		S3|S2	*QGPC(S3|S2)7B*	46	*wPt-5343 –wPt-3533*	3.00	9.49	−0.31
	GMP	S2|S1	*QGMP(S2|S1)7A*	162	*wPt-731311 –Wx-A1*	3.15	9.76	0.95
		S3|S2	*QGMP(S3|S2)3D*	45	*xgpw-4136 –xgwm-3*	4.79	21.06	−1.10
		S4|S3	*QGMP(S4|S3)7A*	80	*wPt-6872 –wPt-0744*	3.31	39.86	1.50
	GApC	S4|S3	*QGApC(S4|S3)3B*	317	*wPt-8206 –wPt-9368*	3.34	14.30	4.09

The abbreviations were the same as those of **Table 1**; S(t)|S(t-1) indicates that the period is S(t-1) to S(t) period.

### 3.3 Comparative analysis of conditional and unconditional QTLs

During grain development, protein synthesis is affected by the cumulative effects of different QTLs/genes. No QTL was detected at all four stages (**Table S5**). From 14 DAA to 21 DAA, 10 QTLs explaining 7.24% to 29.71% of the phenotypic variation had high expression effects, which indicated that genes related to grain protein synthesis are actively expressed. At 21 DAA, two unconditional and conditional QTLs [*QGPC3B*, *QGPC1B*, *QGPC(S3|S2)7A*, and *QGPC(S3|S2)7B*] were significant for protein synthesis. This indicated that the two unconditional QTLs had cumulative genetic effects, while the two conditional QTLs had net genetic effects from 14 DAA to 21 DAA. Two unconditional QTLs, *QGPC2A* and *QGPC3D*, found only at 28 DAA, were not detected in the conditional QTL analysis, which indicated that their cumulative effects were important for protein synthesis during grain development. For GMP synthesis, there were 14 QTLs with high expression effects from 14 DAA to 21 DAA, explaining 5.82% to 39.86% of the variation. Of these, the loci with flanking markers between *wPt-5870* and *wPt-3620* on chromosome 3B included unconditional (*QGMP3B*) and conditional QTLs (*QGMP(S3|S2)3B*), which indicated that this region is important for GMP synthesis with a cumulative genetic effect, while *QGMP(S3|S2)3D* showed a net genetic effect. *QGMP(S2|S1)7A* and *QGMP(S4|S3)7A* also had net genetic effects from 7 DAA to 14 DAA and from 21 DAA to 28 DAA, respectively.

There were two stages with active expression of genes related to the synthesis of amylopectin from 7 DAA to 14 DAA and from 21 DAA to 28 DAA (**Table S5**). Seven unconditional QTLs had cumulative genetic effects, but one conditional QTL, *QGApC(S4|S3)3B*, showed a net genetic effect during GApC synthesis. The two active expression periods for GAsC are the same as those of amylopectin, but 11 QTLs were involved. From 7 DAA to 14 DAA, the unconditional *QTL QGAsC7A* showed a cumulative genetic effect, while *QGAsC(S2|S1)1D* and *QGAsC(S2|S1)6B* had a net genetic effect on GAsC synthesis. From 14 DAA to 21 DAA, there were three unconditional QTLs, *QGAsC1D*, *QGAsC3B*, and *QGAsC7D*, with cumulative genetic effects on GAsC development. However, from 21 DAA to 28 DAA, in addition to the cumulative genetic effects of *QGAsC1B*, *QGAsC3B* and *QGAsC4A*, net genetic effects existed for *QGAsC(S4|S3)1B* and *QGAsC(S4|S3)7A*. Therefore, we know that the expression of genes controlling grain protein and starch contents has obvious temporal and spatial characteristics.

### 3.4 Marker−trait associations of four evaluated traits during grain development

The MTAs at *p* < 10^−4^ were the focus of this study, so these loci are highlighted below. Thirty-one MTAs significantly associated with GPC (*p* < 10^−4^) (**Table 3**; **Figures S5**, **S9**) were identified; they explained 7.62% to 12.97% of the variation and were distributed on chromosomes 3A, 3B, 4A, 4B, 6A, 6B, 7A, and 7B. The SNP locus *Excalibur_c46399_307* on chromosome 6B was detected in two or more environments. Meanwhile, the MTA locus *RAC875_c7060_67* on chromosome 3A had the maximum PVE at 7 DAA in E2 (**Figure S9**).

**Table 3:** Unconditional GWAS of GPC, GMP, GApC, and GAsC during grain development in the nature population (*p* < 10^-4^).

Trait	Period	Env.	Marker	Chromosome	Site	*p*	*R* ^2^ (%)
GPC	S1	E1	*JD_c2026_3994*	3A	90	4.42E-05	8.55
		E2	*BS00075119_51*	3A	15	4.18E-05	8.58
			*D_F5XZDLF02GN47Z_208*	3A	15	4.08E-05	12.73
			*GENE-3869_603*	3A	15	1.54E-05	9.56
			*RAC875_c1666_126*	3A	15	8.99E-06	10.76
			*RAC875_c7060_67*	3A	15	5.72E-07	12.97
			*RAC875_c76948_970*	3A	15	3.79E-06	11.68
			*Tdurum_contig12008_1001*	3A	15	5.60E-07	12.90
			*tplb0050o09_968*	3A	15	3.79E-06	11.68
			*tplb0053a24_2232*	3A	15	7.91E-05	7.83
			*BS00094057_51*	3A	21	3.79E-06	11.68
			*BS00091751_51*	3B	12	5.30E-05	10.99
			*BS00009590_51*	4A	133	1.59E-05	9.44
			*CAP7_c3950_160*	7B	155	2.72E-05	8.90
	S2	E1	*Kukri_c9805_696*	6A	141	8.11E-05	8.20
		E2	*BS00022689_51*	5B	120	8.84E-05	7.62
			*RAC875_c484_1063*	6B	119	2.07E-05	9.11
			*Tdurum_contig12648_389*	6B	119	1.67E-05	9.26
			*Tdurum_contig59339_428*	6B	119	1.67E-05	9.26
			*BS00074289_51*	6B	120	8.44E-05	8.82
			*BS00109878_51*	6B	120	8.44E-05	8.82
			*Kukri_c60966_261*	6B	120	1.67E-05	9.26
			*Tdurum_contig10729_734*	6B	120	1.67E-05	9.26
			*Tdurum_contig10729_986*	6B	120	1.67E-05	9.26
			*RAC875_c17011_717*	6B	121	1.67E-05	9.26
			*TA005876-0602*	6B	121	4.99E-05	8.18
			*Tdurum_contig10729_989*	6B	121	1.67E-05	9.26
			*Tdurum_contig18681_411*	6B	121	1.67E-05	9.26
			*Kukri_rep_c101179_404*	7A	42	7.51E-06	10.06
	S4	E1	*Excalibur_c46399_307*	6B	39	1.07E-05	10.91
		E2	*Excalibur_c46399_307*	6B	39	8.13E-05	8.32
GMP	S1	E2	*BS00075119_51*	3A	15	4.02E-05	8.61
			*D_F5XZDLF02GN47Z_208*	3A	15	4.08E-05	12.77
			*GENE-3869_603*	3A	15	1.72E-05	9.45
			*RAC875_c1666_126*	3A	15	8.90E-06	10.77
			*RAC875_c7060_67*	3A	15	5.63E-07	12.99
			*RAC875_c76948_970*	3A	15	3.81E-06	11.69
			*Tdurum_contig12008_1001*	3A	15	5.48E-07	12.93
			*tplb0050o09_968*	3A	15	3.81E-06	11.69
			*tplb0053a24_2232*	3A	15	7.88E-05	7.84
			*BS00094057_51*	3A	21	3.81E-06	11.69
			BS00091751_51	3B	12	5.64E-05	10.84
			*BS00009590_51*	4A	133	1.98E-05	9.22
			*CAP7_c3950_160*	7B	155	3.07E-05	8.78
	S2	E2	*RFL_Contig1319_507*	3B	67	1.51E-05	9.40
			*Excalibur_c23600_433*	3B	67	1.19E-05	9.62
			*Tdurum_contig42100_381*	3B	67	7.65E-06	10.05
			*Ex_c7101_596*	6B	67	4.95E-05	10.24
	S3	E2	*Tdurum_contig4974_355*	4B	61	2.41E-07	15.69
			*wsnp_Ex_c14654_22713386*	7A	42	2.01E-07	15.30
			*D_contig06359_118*	7D	56	2.92E-07	14.61
	S4	E1	*Tdurum_contig33398_106*	2A	126	1.92E-05	12.49
GApC	S1	E1	*JD_c2026_3994*	3A	90	9.51E-05	7.85
		E2	*BS00091751_51*	3B	12	1.49E-05	10.31
			*BobWhite_c1837_99*	3B	13	1.59E-05	17.86
			*wsnp_Ex_c7478_12792943*	3B	64	7.84E-05	8.12
			*BS00074287_51*	3B	66	2.75E-05	9.20
			*Tdurum_contig12066_126*	5A	83	2.79E-05	9.19
			*Tdurum_contig12066_247*	5A	83	2.79E-05	9.19
			*tplb0027f13_1346*	5A	83	2.79E-05	9.19
			*tplb0027f13_452*	5B	81	1.35E-05	9.95
			*wsnp_Ku_c40334_48581010*	5B	90	2.79E-05	9.19
			*IACX9261*	5B	90	7.31E-05	8.21
			*BobWhite_c48435_165*	5B	90	2.79E-05	9.19
			*Tdurum_contig25513_123*	5B	90	2.86E-05	9.16
			*Tdurum_contig25513_195*	5B	90	2.79E-05	9.19
			*tplb0027f13_1493*	5B	90	2.86E-05	9.16
			*wsnp_Ex_c18639_27511306*	6A	85	6.26E-05	8.35
			*Excalibur_c76268_300*	7A	230	9.71E-05	7.90
			*BS00022550_51*	7B	61	7.36E-06	10.60
			*Tdurum_contig84962_266*	7B	62	1.31E-05	9.97
			*BS00079017_51*	7B	63	7.36E-06	10.60
			*BS00079019_51*	7B	63	7.36E-06	10.60
			*CAP7_c10566_170*	7B	63	6.72E-06	10.68
			*IAAV3391*	7B	63	6.74E-06	11.46
			*IAAV7544*	7B	63	7.36E-06	10.60
			*BobWhite_rep_c64768_264*	7B	63	8.71E-06	10.46
			*BS00022841_51*	7B	63	6.72E-06	10.68
			*Tdurum_contig84962_256*	7B	63	7.36E-06	10.60
	S2	E1	*Kukri_c9805_696*	6A	141	8.82E-05	8.11
		E2	*RAC875_c484_1063*	6B	119	3.73E-05	8.60
			*Tdurum_contig12648_389*	6B	119	3.03E-05	8.75
			*Tdurum_contig59339_428*	6B	119	3.03E-05	8.75
			*Kukri_c60966_261*	6B	120	3.03E-05	8.75
			*Tdurum_contig10729_734*	6B	120	3.03E-05	8.75
			*Tdurum_contig10729_986*	6B	120	3.03E-05	8.75
			*RAC875_c17011_717*	6B	121	3.03E-05	8.75
			*TA005876-0602*	6B	121	7.96E-05	7.79
			*Tdurum_contig10729_989*	6B	121	3.03E-05	8.75
			*Tdurum_contig18681_411*	6B	121	3.03E-05	8.75
			*Kukri_rep_c101179_404*	7A	42	1.73E-05	9.31
	S3	E1	*wsnp_CAP7_c254_138937*	4A	137	6.88E-05	9.48
			*wsnp_CAP7_c254_139077*	4A	137	6.88E-05	9.48
			*IAAV2823*	4A	137	9.73E-05	9.05
			*RAC875_c17185_666*	4A	137	6.88E-05	9.48
			*RFL_Contig3424_931*	4A	137	8.49E-05	9.45
			*BS00021030_51*	4A	137	6.88E-05	9.48
GAsC	S3	E1	*wsnp_Ex_c4436_7981037*	1B	142	2.42E-05	10.89
	S4	E2	*Excalibur_c31194_404*	5A	39	4.70E-05	8.98

The abbreviations were the same as those of **Table 1**.

Twenty-one MTAs were significantly associated with GMP content (*p* < 10^−4^) (**Figures S6**, **S10**) on chromosomes 1A, 3A, 3B, 4A, 4B, 6B, 7A, 7B, and 7D, with 7.84% to 15.69% PVE. Of these, the MTA locus *Tdurum_contig4974_355* on chromosome 4B had the highest PVE (15.69%) at 21 DAA in E2 (**Figure S10**).

Forty-five MTAs for GApC (*p* < 10^−4^) (**Figures S7**, **S11**) were found on chromosomes 3A, 3B, 4A, 5A, 5B, 6A, 6B, 7A, and 7B with 7.35% to 17.86% PVE. Of these, the MTA locus *BobWhite_c1837_99* on chromosome 3B had the highest PVE at 7 DAA in E2 (**Figure S11**).

For GAsC, there were two MTAs (*p* < 10^−4^) (**Figures S8**, **S12**) identified on chromosomes 1B and 5A. Of these, the MTA locus *wsnp_Ex_c4436_7981037* on chromosome 1B had a highest PVE of 10.89% at 21 DAA in E1.

By conditional analysis, at *p* < 10^−4^, a total of 14 MTAs were detected (**Table 4**). Of these, the MTA locus *TA005876-0602* for GPC on chromosome 6B was found at four stages in E2 (**Figure S13**). The MTA locus *Tdurum_contig76213_1156* for GApC on chromosome 4B was identified at four stages in E1 (**Figure S14**), while the MTA locus *wsnp_Ex_c18639_27511306* on chromosome 6A was found at 14 DAA to 28 DAA in E2 (**Figure S15**). However, no MTAs at *p* < 10^−4^ were found under conditional analysis for GMP content and GAsC.

**Table 4:** Conditional GWAS of GPC, GMP, GApC, and GAsC during grain development in the nature population (*p* < 10^-4^).

Trait	Conditional treatment	Env.	Marker	Chromosome	Site	*p*	*R* ^2^ (%)
GPC	S2|S1	E2	*TA005876-0602*	6B	121	6.28E-05	8.21
	S3|S2	E2	*TA005876-0602*	6B	121	6.20E-05	8.22
	S4|S3	E2	*TA005876-0602*	6B	121	6.21E-05	8.22
GApC	S2|S1	E1	*Tdurum_contig76213_1156*	4B	38	8.35E-05	8.03
			*Tdurum_contig82942_681*	4B	38	4.46E-05	8.59
			*tplb0024a16_411*	4B	38	4.46E-05	8.59
	S3|S2	E1	*Tdurum_contig76213_1156*	4B	38	7.96E-05	8.08
			*Tdurum_contig82942_681*	4B	38	4.25E-05	8.64
			*tplb0024a16_411*	4B	38	4.25E-05	8.64
		E2	*wsnp_Ex_c18639_27511306*	6A	85	9.31E-05	7.85
	S4|S3	E1	*Tdurum_contig76213_1156*	4B	38	7.66E-05	8.12
			*Tdurum_contig82942_681*	4B	38	4.09E-05	8.68
			*tplb0024a16_411*	4B	38	4.09E-05	8.68
		E2	*wsnp_Ex_c18639_27511306*	6A	85	9.66E-05	7.81

The abbreviations were the same as those of **Table 2**.

### 3.5 Candidate genes predicted for four evaluated traits during grain development

Markers with high PVE values were selected from loci significantly associated with GMP content, GPC, amylose content, and amylopectin content. Twenty-eight and sixty-nine candidate genes were predicted in *Triticum aestivum* L. (**Table S4**) using QTL mapping and GWAS, respectively. Most of these genes have multiple effects during grain development. Two candidate genes (*TraesCS3B02G159100* and *TraesCS3B02G334400*) (Li S. et al., 2020) for the loci linked with the marker wPt-5870 on chromosome 3B were found to be related to GMP content and GPC, and their functions are related to homeobox-leucine zipper protein 32 (HOX32) activity, DNA-binding transcription factor activity, ATP binding, and protein kinase activity, participating in the biological processes of protein phosphorylation and transcription regulation in *T. aestivum* L. Using the WheatOmics 1.0 (Ma et al., 2021) gene expression level database to screen these two genes (**Table S7**), we found that the expression levels of them were higher at 20 days after flowering, which is the same in our results (S3 development stage). For the conditional loci linked with the marker wPt-0744 related to GMP content, GPC, and GAsC, 13 candidate genes were predicted, but no genes were found on chromosome 7A. Two predictive genes, *TraesCS6A02G298100* (Andleeb et al., 2023; Teng et al., 2022) and *TraesCS5A02G326100*, encode glutamine synthetase and cysteine protease, respectively, and influence protein synthesis during grain development. The functions of the residue candidate genes are mainly related to ATP binding, protein serine/threonine kinase activity, dipeptide or tripeptide transmembrane transporter activity, histone deacetylase activity, NAD+ binding, and transferase activity. These two genes have higher expression levels in S3 and S2 stages, respectively, by using the WheatOmics 1.0 gene expression level database analysis. The candidate gene *TraesCS2A02G012600* is involved in carbohydrate metabolic processes. It had the highest expression level at 20 days after flowering (S3 development stage) using the WheatOmics 1.0 (Ma et al., 2021) gene expression level database. There are three candidate genes (*TraesCS7A02G070100*, *TraesCS4A02G418200*, and *TraesCS7D02G064300*) predicted for *Wx-A1* on chromosomes 7A, 4A, and 7D (Liu et al., 2020; Tian et al., 2022; Kaushik et al., 2020; Guo et al., 2020) related to amylose and GPC, encoding starch synthase, chloroplastic/amyloplastic starch synthase, and granule-bound starch synthase. The expression levels of these three genes were higher at 10 days after flowering (S2 development stage) by using the WheatOmics 1.0 gene expression level database. Their functions are glycan biosynthesis, starch biosynthesis, and glycogen (starch) synthase activity, which confirms the accuracy of the results. In addition, one candidate gene was predicted for GMP content that is involved in cyclic pyranopterin monophosphate synthase activity. 999[JFATwo candidate genes (*TraesCS2A02G238400* and *TraesCS2A02G537100*) (Grzesiak et al., 2019) for GPC are related to zeta-carotene desaturase and superoxide dismutase. The expression levels of these two genes were higher at 10 and 20 days after flowering (S2 development stage), respectively. Five candidate genes were predicted for GApC. Among the 69 candidate genes associated with important SNP sites, there were 39 candidate genes related to GMP content and GPC, mainly involving 11 chromosomes, especially 22 genes on 3A and 3B. Most of their functions are related to acid–amino acid ligase activity, myosin phosphatase activity, protein serine/threonine phosphatase activity, proton transmembrane transporter activity, GTPase activator activity, amino acid binding, aspartate carbamoyl transferase activity, phosphatidylinositol binding, and ubiquitin binding, participating in protein synthesis in wheat.

The SNP marker *BS00091751_51* was associated with GApC, GMP content, and GPC during grain development. Eight candidate genes were predicted on chromosomes 3A, 3B, and 1B. The two genes *TraesCS3A02G320800* (High expression level during S3 period) and *TraesCS3A02G112600* (High expression level during S4 period), encode glucose-induced degradation protein 8 homologue and UBC core domain-containing protein, respectively. The candidate gene *TraesCS1B02G368500* (High expression level during S4 period) has glycogen (starch) synthase activity and is involved in starch biosynthetic processes. Six candidate genes for SNP marker *Kukri_rep_c101179_404* were also predicted to be associated with GApC, GMP content, and GPC. Of these, the candidate gene *TraesCS3B02G345000* (High expression level around the S2 period) is related to *BS00091751_51*, not *Kukri_rep_c101179_404*. Although no genes were found on chromosome 7A, two genes (High expression level around the S3 period) with unknown functions (*TraesCS7B02G305600* and *TraesCS7B02G305500*) were screened on chromosome 7B. The candidate gene *TraesCS3B02G021900* is involved in glycosyltransferase activity and pentosyltransferase activity.

Five candidate genes for the *Excalibur_c46399_307* marker related to GPC were found, and their functions were related to DNA binding, GTP binding, and GTPase activity; they encoded NAC transcription factors and ADP-ribosylation factors. There were 17 candidate genes predicted for GMP content mainly on chromosomes 3B, 2D, and 2A associated with SNP markers *Tdurum_contig42100_381*, *Tdurum_contig4974_355*, and *wsnp_Ex_c14654_22713386*. The gene *TraesCS2D02G261300* (High expression level during the S1 or S2 period) has glutaminyl-tRNA synthase (glutamine-hydrolysing) activity, encoding glutamyl-tRNA (Gln) amidotransferase subunit B. The gene *TraesCS3B02G254200* (High expression level during the S1 or S2 period) has glycosyltransferase activity, while the genes *TraesCS2D02G308600* (Guo et al., 2020) (High expression level during the S1 or S2 period) and *TraesCS1B02G368500* (High expression level during S4 period) are involved in starch biosynthesis. We found that some important genes on chromosome 3B were related to protein and starch synthesis during grain development using two mapping methods. These candidate genes may be important for GMP content, GPC, amylose content, and amylopectin content during grain development, and their functions will be further identified in future research.

## 4 Discussion

### 4.1 Accumulation changes of four quality characters during grain development

Previous studies showed that the accumulation changes in GPC presented a trend of “high–low–high” during grain filling (Zhang and Xu, 1995). In this study, the dynamic change in protein content also showed a “high–low–high” trend during the grain development of wheat, which was less affected by the environment and genetic background. The decrease in the total crude protein content observed during grain development might be due to extensive non- water-soluble carbohydrate accumulation, mainly starch. This decrease was followed by a vast accumulation of crude protein, which was mentioned before in other studies during the grain filling and maturation phases, which might result from the final desiccation prior to maturation (Panozzo et al., 2001; Verspreet et al., 2013). A study showed that GMP content changed from 12 DAA to the mature stage and that the accumulation amount remained relatively stable at a low level before 20 DAA, and then gradually increased from 20 DAA to the mature stage, during grain filling (Huang et al., 2007). In this study, the dynamic change in GMP content showed a trend of “high–low–high” from 7 DAA to the mature stage. This was similar to the accumulation trend of GPC. However, there have been few studies on the accumulation changes in GApC and GAsC during wheat grain development, and most of them focused on the key enzyme activity of starch synthesis in grain development. The activities of soluble starch synthase and starch granule-bound starch synthetase both first increased and then decreased during development (Wang et al., 2007), but they reached maximum activity at different times. This was similar to the accumulation changes in amylose content and amylopectin content. These results provide a reference for the study of protein content in wheat at certain developmental stages and is an important prerequisite for the study of how amylase affects starch content in wheat grains.

### 4.2 Major chromosomes for grain protein, GMP, amylose, and amylopectin synthesis during grain development

Previous studies have shown that most chromosomes are related to GPC. Using the RIL population, QTLs were identified on chromosomes 2A, 2B, 2D, 3D, 4A, 6B, 7A, and 7D (Prasad et al., 2003), and chromosomes 6B and 7A had major QTLs with flanking markers *Xgwm133, Xgwm889*, and *Xgwm1171.* Meanwhile, some important QTLs for GPC were detected on chromosomes 3B, 6B, and 7B (Chee et al., 2001; Sun et al., 2007; Bordes et al., 2011). In addition, previous researchers also found QTLs for GMP content on chromosomes 3B, 5B, 6B, and 7B (Li et al., 2007; Sun et al., 2007). In this study, both QTLs and MTAs for GPC were identified on chromosomes 3B, 7A, and 7B from 14 DAA to 21 DAA using QTL mapping and GWAS. The loci on chromosomes 3B, 7A, and 7B were closely linked to the markers *wPt-5870–wPt-3620* and *BS00091751_51*, *Wx-A1* and *Kukri_rep_c101179_404*, and *wPt-5343* and *CAP7_c3950_160*, respectively. On chromosome 6B, there was an important SNP marker, *TA005876-0602*, linked to GPC at four stages. However, for GMP content during grain development, loci on chromosomes 2A, 3B, 4A, 7A, and 7B were found using both QTL mapping and GWAS. Some important linked markers were identified, such as wPt-3896 and *Tdurum_contig33398_10* on chromosome 2A, *wPt-3620 and BS00091751_51* on chromosome 3B, *wPt-731137* and *BS00009590_51* on chromosome 4A, *Wx-A1* and *wsnp_Ex_c14654_22713386* on chromosome 7A, and *wPt-3533* and *CAP7_c3950_160* on chromosome 7B. By comparison with previous studies, chromosomes 3B and 7B played important roles in protein and GMP synthesis during grain development. There was one important new locus on chromosome 3B involved in both protein and GMP synthesis.

Starch content affects not only wheat yield but also quality, which is influenced by amylose and amylopectin synthesis. Previous studies detected more than 20 QTLs for starch traits, most of which were found on chromosomes 3D, 6B, and 7B (Sun et al., 2007). The QTLs for GApC were identified on chromosomes 1B, 3A, 3B, and 5D (Tian et al., 2011). Although amylose synthesis is mainly affected by waxy genes, other chromosomes may also affect amylose content because it is a quantitative trait. There was one additive QTL found on chromosome 6B with flanking markers between Cau170 and xgwm234 (Wang et al., 2007). In the present study, there were 11 (2A, 3A, 3B, 4A, 5A, 5B, 6A, 6B, 7A, and 7B) and eight chromosomes (1B, 1D, 3B, 4A, 5A, 6B, 7A, and 7D) involved in amylopectin and amylose synthesis during grain development. For amylopectin content, the loci on chromosomes 3B and 4A were detected by both QTL mapping and GWAS, while for amylose, only the loci on chromosome 1B were detected by both methods. Most interestingly, the loci were identified on chromosomes 4A, 7A, and 7D close to three waxy genes. Of these, the loci on chromosome 4A were involved in not only amylose synthesis but also amylopectin synthesis during grain development. These results indicated that the results were accurate and reliable. However, by comparing the physical location of the important loci between QTL mapping GWAS, we unfortunately found that there was no loci on the same interval physical positions (**Table S6**).

### 4.3 The role of chromosome 3B in protein and starch synthesis during grain development

All four chromosomes (3B, 4A, 6B, and 7A) were simultaneously associated with protein, GMP, amylopectin, and amylose synthesis during grain development. In particular, the region of chromosome 3B with flanking markers between wPt-5870 and wPt-3620 was shown to be important for protein and starch synthesis. This chromosome region was involved in protein synthesis from 14 DAA to 21 DAA, GMP synthesis from 0 to 7 DAA, and amylose synthesis from 14 DAA to 21 DAA. In addition, from 0 to 7 DAA and from 21 DAA to 28 DAA, the other chromosome region of 3B was involved in amylopectin synthesis. Therefore, from 0 to 7 DAA, chromosome 3B was mainly involved in the synthesis of GMP and amylopectin; from 14 DAA to 21 DAA, it was mainly involved in protein and GMP synthesis; and from 21 DAA to 28 DAA, it was mainly involved in amylose and amylopectin synthesis.

### 4.4 Candidate genes during protein and starch accumulation in wheat grain filling

Previous studies have shown that protein accumulation during grain development is affected by regulation at the transcriptional level and post-transcriptional level, which involves some transcription factors (TFs) regulating prolamin genes, such as MYB TFs, AP2, and NAC TFs, and some important protein modifications, such as N-glycosylation, folding, and assembly of proteins, and protein bodies (Diaz et al., 2002; Rubio-Somoza et al., 2006; Zhang et al., 2019), while starch synthesis in wheat is affected by a series of enzymes, including ADP-glucose pyrophosphorylase, sucrose synthase, granule bound starch synthase, starch synthase, starch branching enzyme, and starch debranching enzyme (Kumar et al., 2018). Meanwhile, the interactions between opaque-2 and prolamin-box binding factor can regulate the gene networks for starch and protein biosynthesis in maize (Zhang et al., 2017). In rice, there is also a similar interaction between bZIP and DNA binding with one finger TFs (Kim et al., 2017). However, one storage protein activator, *TaSPA-B*, plays an important role in starch and protein biosynthesis by regulating a complex gene network in wheat (Guo et al., 2020). In our study, we also found some candidate genes related to not only protein synthesis but also starch synthesis during grain development. For example, *TraesCS6A02G298100* (Andleeb et al., 2023) and *TraesCS5A02G326100* function in ATP binding, glutamate-ammonia ligase activity, and cysteine-type peptidase activity, encoding glutamine synthetase and cysteine protease, respectively, which have been shown to play important roles in protein synthesis in previous studies (Gadaleta et al., 2011). Meanwhile, some identified genes have functions of glycosyltransferase activity, GTPase activator activity, 1,4-alpha-glucan branching enzyme activity, GTPase activity, etc., which are all involved in starch synthesis. Interestingly, we also identified genes that encode NAC transcription factors, *TraesCS6B02G075200* (Saini et al., 2021), *TraesCS5D02G059700* (Grzesiak et al., 2019), *TraesCS5B02G054200* (Vranic et al., 2022), and *TraesCS5A02G049100*. The same two genes shown to be involved in starch synthesis in our study, *TraesCS7D02G117800* (*SSI*) (Tian et al., 2022) and *TraesCS2D02G308600* (*GBE1*, *SBE*), were also reported in Guo’s study (Guo et al., 2020). In addition, the gene network was constructed based on these predicted QTL/genes to verify the importance and connection among predicted genes (**Table S7**). We found that there were four clusters for predicted genes, which were related to grain hardness, grain length, grain size, grain weight, and starch grain. These indicated that they participated in the synthesis of protein and starch (**Figure S16**). However, a total of 67 candidate genes have been screened, and their multiple mechanisms of protein and starch synthesis during wheat grain development need to be studied further in the future.

## 5 Conclusion

We detected 10 unconditional major QTLs, 7 conditional major QTLs, and 36 unconditional major MTAs for four evaluated traits during grain development. Pleiotropic QTLs/MTAs have been identified. Four chromosomes were mainly involved in the development of protein, GMP, amylopectin, and amylose synthesis. Twenty-eight and 69 important genes for major loci were predicted, respectively, and most of them have multiple effects on protein and starch synthesis during grain development. Overall, our study provides novel insights that increase our understanding of the genetic information of protein and starch synthesis during grain development, and will help improve our understanding of the regulatory network between grain protein and starch in wheat breeding programs.

## Data availability statement

The original contributions presented in the study are included in the article/**Supplementary Material**. Further inquiries can be directed to the corresponding authors.

## Author contributions

ZD designed and revised this paper; JT constructed the populations; YG, GW, and DW analyzed the data and wrote the manuscript; XG, SC, and HY investigated and analyzed the phenotypic data; KJ, HH, and CW analyzed and screened the candidate genes; JC, YB, and WZ reviewed this paper; all authors have read and approved this manuscript.
