# Supplementary material for: Genetic dissection of protein and starch during wheat grain development using QTL mapping and GWAS
Source: Front Plant Sci. 2023 Jun 12;14:1189887. doi: 10.3389/fpls.2023.1189887 (PMC10291175; doi:10.3389/fpls.2023.1189887)
Supplement: Supplementary file 1 [file Table_1.docx]

Table S1 The phenotypic varation of the content of protein, GMP, amylopectin and amylose during grain development in RIL populations and their parents in different years

| Env. | Trait | Period | Parents | | RIIL Population | | | | | |
| --- | --- | --- | --- | --- | --- | --- | --- | --- | --- | --- |
|  |  |  | NM1 | GC8901 | Mean | Mixmum | Minmum | S.D. | Skewness | Kurtosis |
| E1 | GPC(%) | S1 | 15.34 | 18.23 | 16.69 | 22.14 | 11.15 | 2.25 | -0.29 | -0.52 |
|  |  | S2 | 14.09 | 15.77 | 14.66 | 17.35 | 10.96 | 1.06 | -0.02 | 0.88 |
|  |  | S3 | 13.21 | 15.16 | 13.25 | 17.26 | 10.34 | 1.01 | 0.52 | 1.49 |
|  |  | S4 | 13.38 | 16.67 | 14.14 | 16.46 | 9.42 | 0.93 | -0.61 | 2.59 |
|  | GMP(mg/g) | S1 | 11.45 | 13.98 | 15.36 | 25.26 | 5.01 | 3.00 | -0.04 | 1.00 |
|  |  | S2 | 11.10 | 12.56 | 11.64 | 18.32 | 2.42 | 2.73 | -0.22 | 0.18 |
|  |  | S3 | 10.67 | 11.99 | 10.52 | 19.05 | 0.76 | 2.32 | -1.04 | 4.02 |
|  |  | S4 | 10.90 | 12.14 | 11.66 | 19.46 | 2.03 | 2.26 | -0.42 | 1.63 |
|  | GApC(%) | S1 | 38.03 | 59.37 | 45.91 | 64.13 | 20.24 | 9.39 | -0.60 | 0.44 |
|  |  | S2 | 45.18 | 62.03 | 54.02 | 67.57 | 29.82 | 7.82 | -0.61 | -0.15 |
|  |  | S3 | 56.10 | 58.23 | 53.33 | 74.69 | 26.30 | 7.94 | -0.49 | 0.31 |
|  |  | S4 | 60.29 | 65.37 | 61.01 | 77.50 | 38.91 | 7.35 | -0.35 | -0.13 |
|  | GAsC(%) | S1 | 1.27 | 6.60 | 5.97 | 14.13 | 0.00 | 3.31 | -0.31 | -0.64 |
|  |  | S2 | 1.68 | 4.99 | 5.61 | 9.74 | 0.51 | 1.53 | -0.53 | 0.70 |
|  |  | S3 | 2.30 | 6.56 | 5.81 | 9.53 | 1.36 | 1.26 | -0.28 | 0.63 |
|  |  | S4 | 3.11 | 7.93 | 6.74 | 10.43 | 0.73 | 1.44 | -0.65 | 1.63 |
| E2 | GPC(%) | S1 | 12.71 | 14.88 | 16.06 | 23.48 | 12.63 | 2.22 | 0.78 | 0.23 |
|  |  | S2 | 11.48 | 14.30 | 14.49 | 18.94 | 10.38 | 1.71 | 0.32 | -0.35 |
|  |  | S3 | 13.71 | 15.54 | 14.92 | 18.47 | 12.23 | 1.05 | 0.31 | 0.22 |
|  |  | S4 | 15.27 | 15.74 | 15.28 | 18.14 | 12.09 | 1.05 | -0.07 | 0.39 |
|  | GMP(mg/g) | S1 | 8.39 | 8.85 | 11.93 | 23.80 | 3.24 | 3.67 | 0.46 | -0.19 |
|  |  | S2 | 10.87 | 12.88 | 10.38 | 17.51 | 0.12 | 3.07 | -0.25 | 0.03 |
|  |  | S3 | 8.87 | 10.63 | 10.94 | 18.32 | 4.26 | 2.47 | -0.19 | 0.44 |
|  |  | S4 | 8.33 | 13.10 | 13.27 | 21.19 | 6.63 | 2.20 | 0.17 | 0.61 |
|  | GApC(%) | S1 | 18.57 | 27.10 | 14.69 | 38.55 | 0.08 | 9.39 | 0.51 | -0.56 |
|  |  | S2 | 34.19 | 35.01 | 31.50 | 55.77 | 4.17 | 9.47 | 0.23 | 0.07 |
|  |  | S3 | 21.71 | 46.58 | 40.71 | 57.94 | 15.20 | 8.57 | -0.37 | 0.11 |
|  |  | S4 | 43.77 | 48.18 | 37.64 | 59.54 | 9.43 | 9.72 | -0.60 | 0.55 |
|  | GAsC(%) | S1 | - | - | - | - | - | - | - | - |
|  |  | S2 | - | - | - | - | - | - | - | - |
|  |  | S3 | 2.14 | 2.10 | 3.61 | 7.42 | 0.00 | 1.33 | 0.08 | 0.89 |
|  |  | S4 | 2.16 | 5.04 | 3.10 | 11.48 | 0.00 | 1.88 | 1.03 | 2.84 |

Note: Env. Indicates Environment, S.D. means Standard deviation, E1 means 2016-2017, E2 means 2017-2018, S1 means 7 days after flowering, S2 means 14 days after flowering, S3 means 21 days after flowering, and S4 means 28 days after flowering, "-" means not detected.The same as follow.

Table S2 The phenotypic variation of the content of protein, GMP, amylopectin and amylose during grain development in nature populations in different years

| Env. | Trait | Period | Mean | Miximum | Minmum | S.D. | Skewness | Kurtosis |
| --- | --- | --- | --- | --- | --- | --- | --- | --- |
| E1 | GPC(%) | S1 | 18.16 | 25.14 | 11.37 | 3.30 | -0.12 | -0.91 |
|  |  | S2 | 14.14 | 19.25 | 11.09 | 1.24 | 0.92 | 2.58 |
|  |  | S3 | 13.18 | 17.25 | 9.90 | 1.32 | 0.18 | 0.05 |
|  |  | S4 | 13.27 | 17.15 | 10.58 | 1.20 | 0.30 | 0.02 |
|  | GMP(mg/g) | S1 | 14.98 | 24.59 | 1.28 | 5.85 | -0.29 | -0.76 |
|  |  | S2 | 12.16 | 20.52 | 1.10 | 3.90 | -0.21 | -0.28 |
|  |  | S3 | 9.75 | 15.11 | 3.90 | 2.03 | 0.04 | -0.19 |
|  |  | S4 | 10.24 | 16.62 | 3.20 | 2.04 | -0.25 | 0.64 |
|  | GApC(%) | S1 | 31.25 | 57.61 | 7.38 | 13.39 | 0.31 | -1.07 |
|  |  | S2 | 42.92 | 57.14 | 24.02 | 6.73 | -0.44 | -0.14 |
|  |  | S3 | 57.47 | 72.17 | 36.07 | 8.83 | -0.50 | -0.73 |
|  |  | S4 | 65.96 | 77.36 | 45.02 | 6.03 | -0.77 | 0.82 |
|  | GAsC(%) | S1 | 3.30 | 10.02 | 0.03 | 2.05 | 0.90 | 0.45 |
|  |  | S2 | 5.72 | 10.07 | 0.56 | 2.09 | -0.26 | -0.45 |
|  |  | S3 | 7.66 | 13.63 | 2.02 | 2.35 | 0.30 | -0.49 |
|  |  | S4 | 10.21 | 13.82 | 5.00 | 1.55 | -0.36 | 0.48 |
| E2 | GPC(%) | S1 | 15.16 | 20.72 | 12.46 | 1.38 | 1.45 | 2.76 |
|  |  | S2 | 13.58 | 16.36 | 3.46 | 1.46 | -2.48 | 13.49 |
|  |  | S3 | 14.10 | 17.71 | 10.90 | 1.31 | 0.01 | -0.33 |
|  |  | S4 | 15.14 | 18.26 | 11.01 | 1.28 | -0.13 | -0.07 |
|  | GMP(mg/g) | S1 | 9.07 | 24.97 | 1.83 | 3.21 | 0.89 | 3.09 |
|  |  | S2 | 6.77 | 15.29 | 1.35 | 2.88 | 0.25 | -0.42 |
|  |  | S3 | 8.92 | 15.07 | 3.60 | 2.07 | -0.24 | -0.14 |
|  |  | S4 | 10.14 | 20.26 | 4.83 | 2.49 | 0.85 | 2.07 |
|  | GApC(%) | S1 | 17.41 | 50.91 | 1.66 | 10.60 | 0.94 | 0.64 |
|  |  | S2 | 46.75 | 68.43 | 9.11 | 9.39 | -0.33 | 0.67 |
|  |  | S3 | 38.05 | 68.73 | 20.21 | 8.81 | 0.83 | 1.75 |
|  |  | S4 | 39.23 | 74.70 | 23.64 | 8.65 | 1.52 | 3.66 |
|  | GAsC(%) | S1 | - | - | - | - | - | - |
|  |  | S2 | - | - | - | - | - | - |
|  |  | S3 | 2.54 | 10.40 | 0.10 | 1.63 | 2.02 | 6.04 |
|  |  | S4 | 2.61 | 10.75 | 0.16 | 1.82 | 2.55 | 8.45 |

Note:The abbreviations were the same as those of the Table S1.

Table S3-1 The correlation analysis of protein and starch related traits of the RIL population in E1 environment

| Trait | | _GPC | | | | GMP | | | | GApC | | | | GAsC | | | |
| --- | --- | --- | --- | --- | --- | --- | --- | --- | --- | --- | --- | --- | --- | --- | --- | --- | --- |
|  |  | S1 | S2 | S3 | S4 | S1 | S2 | S3 | S4 | S1 | S2 | S3 | S4 | S1 | S2 | S3 | S4 |
| GPC | S1 | 1 |  |  |  |  |  |  |  |  |  |  |  |  |  |  |  |
|  | S2 | 0.139* | 1 |  |  |  |  |  |  |  |  |  |  |  |  |  |  |
|  | S3 | -0.140* | 0.119* | 1 |  |  |  |  |  |  |  |  |  |  |  |  |  |
|  | S4 | 0.055 | 0.229** | 0.396** | 1 |  |  |  |  |  |  |  |  |  |  |  |  |
| GMP | S1 | 0.590** | 0.146* | 0.048 | 0.112 | 1 |  |  |  |  |  |  |  |  |  |  |  |
|  | S2 | -0.017 | 0.272** | -0.11 | -0.022 | -0.033 | 1 |  |  |  |  |  |  |  |  |  |  |
|  | S3 | -0.024 | -0.003 | 0.073 | 0.193** | 0.077 | 0.150* | 1 |  |  |  |  |  |  |  |  |  |
|  | S4 | 0.006 | 0.02 | 0.093 | 0.445** | -0.132* | 0.351** | 0.132* | 1 |  |  |  |  |  |  |  |  |
| GApC | S1 | -0.162* | 0.202** | -0.034 | -0.01 | -0.076 | 0.024 | 0.189** | 0.027 | 1 |  |  |  |  |  |  |  |
|  | S2 | -0.151* | 0.093 | 0.073 | 0.004 | -0.069 | 0.014 | -0.028 | 0.144* | 0.314** | 1 |  |  |  |  |  |  |
|  | S3 | -0.033 | -0.021 | 0.027 | -0.031 | -0.043 | -0.133* | -0.187** | 0.062 | 0.103 | 0.063 | 1 |  |  |  |  |  |
|  | S4 | -0.021 | 0.111 | -0.022 | 0.043 | 0.124* | -0.099 | 0.011 | 0.061 | 0.189** | 0.192** | 0.377** | 1 |  |  |  |  |
| GAsC | S1 | -0.613** | 0.077 | 0.103 | -0.031 | -0.289** | 0.117 | 0.061 | -0.074 | 0.383** | 0.160* | -0.006 | 0.142* | 1 |  |  |  |
|  | S2 | 0.002 | -0.201** | -0.016 | -0.038 | 0.048 | 0.460** | 0.06 | 0.311** | 0.068 | 0.294** | 0.026 | 0.130* | 0.131* | 1 |  |  |
|  | S3 | -0.144* | -0.190** | -.123* | 0.098 | -0.138* | -0.071 | 0.157* | 0.156* | 0.223** | 0.247** | 0.453** | 0.170** | 0.04 | 0.219** | 1 |  |
|  | S4 | -0.238** | 0.039 | 0.025 | -0.104 | -0.081 | -0.086 | 0.007 | 0.181** | 0.373** | 0.517** | 0.197** | 0.469** | 0.205** | 0.231** | 0.293** | 1 |

Note: The abbreviations were the same as those of the Table 2; ** indicates a significant correlation at the 0.01 level (one side); * indicates a significant correlation at the 0.05 level (one side).

Table S3-2 The correlation analysis of protein and starch related traits of the RIL population in E2 environment

| Trait | | GPC | | | | GMP | | | | GApC | | | | GAsC | |
| --- | --- | --- | --- | --- | --- | --- | --- | --- | --- | --- | --- | --- | --- | --- | --- |
|  |  | S1 | S2 | S3 | S4 | S1 | S2 | S3 | S4 | S1 | S2 | S3 | S4 | S3 | S4 |
| GPC | S1 | 1 |  |  |  |  |  |  |  |  |  |  |  |  |  |
|  | S2 | 0.131* | 1 |  |  |  |  |  |  |  |  |  |  |  |  |
|  | S3 | 0.038 | 0.266** | 1 |  |  |  |  |  |  |  |  |  |  |  |
|  | S4 | -0.008 | 0.201** | 0.584** | 1 |  |  |  |  |  |  |  |  |  |  |
| GMP | S1 | 0.762** | 0.108 | 0.074 | -0.016 | 1 |  |  |  |  |  |  |  |  |  |
|  | S2 | 0.074 | 0.574** | 0.062 | 0.129* | 0.251** | 1 |  |  |  |  |  |  |  |  |
|  | S3 | -0.298** | -0.012 | 0.246** | 0.391** | -0.222** | 0.162* | 1 |  |  |  |  |  |  |  |
|  | S4 | 0.157* | 0.160* | 0.310** | 0.212** | 0.044 | -0.118 | -0.086 | 1 |  |  |  |  |  |  |
| GApC | S1 | -0.237** | 0.082 | 0.013 | 0.097 | -0.379** | -0.190* | 0.224** | 0.125 | 1 |  |  |  |  |  |
|  | S2 | -0.046 | -0.360** | -0.036 | 0.068 | -0.091 | -0.362** | 0.170* | 0.008 | 0.208** | 1 |  |  |  |  |
|  | S3 | 0.274** | 0.12 | 0.056 | -0.147* | 0.247** | -0.109 | -0.379** | 0.332** | 0.074 | 0.064 | 1 |  |  |  |
|  | S4 | 0.067 | -0.233** | -0.186** | 0.007 | 0.150* | -0.094 | 0.041 | 0.190** | 0.01 | 0.204** | 0.247** | 1 |  |  |
| GAsC | S3 | -0.049 | -0.215** | -0.226** | -0.177** | -0.053 | -0.265** | -0.091 | 0.236** | 0.268** | 0.263** | 0.533** | 0.391** | 1 |  |
|  | S4 | 0.142* | -0.126* | -0.215** | -0.249** | 0.045 | -0.282** | -0.216** | 0.446** | 0.186* | 0.278** | 0.341** | 0.627** | 0.600** | 1 |

Note: The abbreviations were the same as those of the Table 2;** indicates a significant correlation at the 0.01 level (one side); * indicates a significant correlation at the 0.05 level (one side).

Table S3-3 The correlation analysis of protein and starch related traits of the nature population in E1 environment

| Trait | | GPC | | | | GMP | | | | GApC | | | | GAsC | | | |
| --- | --- | --- | --- | --- | --- | --- | --- | --- | --- | --- | --- | --- | --- | --- | --- | --- | --- |
|  |  | S1 | S2 | S3 | S4 | S1 | S2 | S3 | S4 | S1 | S2 | S3 | S4 | S1 | S2 | S3 | S4 |
| GPC | S1 | 1 |  |  |  |  |  |  |  |  |  |  |  |  |  |  |  |
|  | S2 | 0.218** | 1 |  |  |  |  |  |  |  |  |  |  |  |  |  |  |
|  | S3 | 0.099 | 0.275** | 1 |  |  |  |  |  |  |  |  |  |  |  |  |  |
|  | S4 | 0.226** | 0.472** | .409** | 1 |  |  |  |  |  |  |  |  |  |  |  |  |
| GMP | S1 | 0.813** | 0.076 | -0.039 | 0.067 | 1 |  |  |  |  |  |  |  |  |  |  |  |
|  | S2 | 0.074 | 0.399** | -0.105 | 0.135* | 0.146* | 1 |  |  |  |  |  |  |  |  |  |  |
|  | S3 | -0.095 | 0.310** | 0.042 | 0.395** | -0.13 | 0.336** | 1 |  |  |  |  |  |  |  |  |  |
|  | S4 | 0.048 | 0.259** | 0.141* | 0.219** | -0.031 | 0.08 | 0.172* | 1 |  |  |  |  |  |  |  |  |
| GApC | S1 | 0.555** | 0.105 | 0.025 | 0.098 | 0.445** | 0.004 | -0.135 | -0.011 | 1 |  |  |  |  |  |  |  |
|  | S2 | 0.053 | 0.118 | 0.04 | 0.068 | 0.170* | 0.143* | 0.048 | 0.160* | -0.088 | 1 |  |  |  |  |  |  |
|  | S3 | 0.006 | 0.108 | 0.204** | 0.132* | -0.056 | 0.115 | .341** | 0.018 | -0.102 | 0.161* | 1 |  |  |  |  |  |
|  | S4 | -0.071 | 0.102 | -0.015 | 0.08 | -0.068 | 0.02 | 0.148* | 0.071 | -0.229** | 0.378** | 0.210** | 1 |  |  |  |  |
| GAsC | S1 | -0.573** | -0.053 | 0.093 | -0.142* | -0.463** | 0.01 | 0.004 | 0.035 | -0.131 | 0.116 | 0.018 | 0.104 | 1 |  |  |  |
|  | S2 | -0.02 | -0.404** | -0.280** | -0.138* | 0.205** | 0.310** | -0.002 | -0.065 | -0.142 | 0.548** | -0.05 | 0.215** | 0.008 | 1 |  |  |
|  | S3 | -0.056 | -0.022 | -0.473** | -0.016 | -0.052 | 0.189* | 0.463** | -0.001 | -0.082 | -0.039 | 0.467** | 0.058 | -0.012 | 0.11 | 1 |  |
|  | S4 | -0.115 | -0.313** | -0.178** | -0.521** | -0.01 | -.222** | -0.278** | 0.147* | -0.104 | 0.127* | -0.126 | 0.298** | 0.130* | 0.210** | -0.019 | 1 |

Note:The abbreviations were the same as those of the Table 2;** indicates a significant correlation at the 0.01 level (one side); * indicates a significant correlation at the 0.05 level (one side).

Table S3-4 The correlation analysis of protein and starch related traits of the nature population in E2 environment

| Trait | | GPC | | | | GMP | | | | GApC | | | | GAsC | |
| --- | --- | --- | --- | --- | --- | --- | --- | --- | --- | --- | --- | --- | --- | --- | --- |
|  |  | S1 | S2 | S3 | S4 | S1 | S2 | S3 | S4 | S1 | S2 | S3 | S4 | S3 | S4 |
| GPC | S1 | 1 |  |  |  |  |  |  |  |  |  |  |  |  |  |
|  | S2 | 0.382** | 1 |  |  |  |  |  |  |  |  |  |  |  |  |
|  | S3 | 0.01 | 0.363** | 1 |  |  |  |  |  |  |  |  |  |  |  |
|  | S4 | -0.026 | 0.217** | 0.707** | 1 |  |  |  |  |  |  |  |  |  |  |
| GMP | S1 | 0.520** | 0.336** | -0.101 | -0.067 | 1 |  |  |  |  |  |  |  |  |  |
|  | S2 | 0.448** | 0.500** | 0.02 | -0.082 | 0.523** | 1 |  |  |  |  |  |  |  |  |
|  | S3 | -0.002 | 0.211** | 0.359** | 0.219** | -0.087 | 0.07 | 1 |  |  |  |  |  |  |  |
|  | S4 | 0.016 | 0.067 | 0.297** | 0.270** | -0.016 | 0.018 | 0.350** | 1 |  |  |  |  |  |  |
| GApC | S1 | 0.03 | -0.212** | 0.169* | 0.228** | -0.165* | -0.08 | 0.042 | 0.239** | 1 |  |  |  |  |  |
|  | S2 | -0.085 | -0.058 | 0.088 | 0.116 | -0.169* | -0.353** | 0.101 | 0.063 | 0.146* | 1 |  |  |  |  |
|  | S3 | 0.241** | -0.114 | -0.041 | -0.173** | 0.149* | 0.104 | 0.097 | 0.241** | 0.292** | 0.207** | 1 |  |  |  |
|  | S4 | 0.141* | 0.093 | -0.011 | -0.007 | 0.286** | 0.249** | -0.09 | 0.360** | 0.241** | 0.05 | 0.533** | 1 |  |  |
| GAsC | S3 | 0.041 | -0.084 | -0.264** | -0.282** | 0.137* | 0.127 | 0.081 | 0.317** | 0.151* | 0.216** | 0.615** | 0.524** | 1 |  |
|  | S4 | 0.086 | -0.075 | -0.181** | -0.266** | 0.193** | 0.178* | -0.052 | 0.466** | 0.174* | 0.083 | 0.521** | 0.635** | 0.754** | 1 |

Note:The abbreviations were the same as those of the Table 2;** indicates a significant correlation at the 0.01 level (one side); * indicates a significant correlation at the 0.05 level (one side).


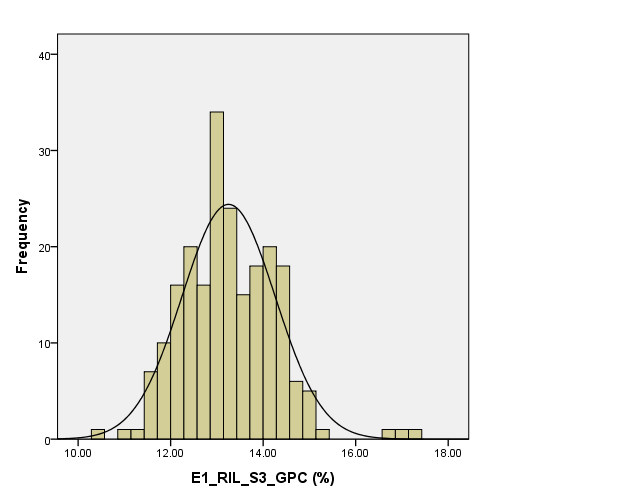

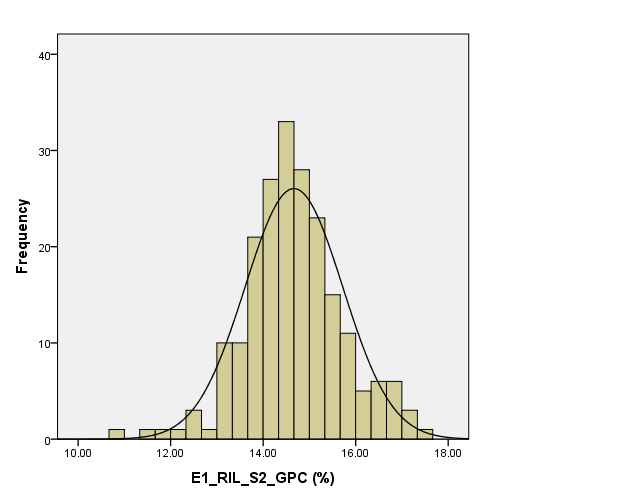

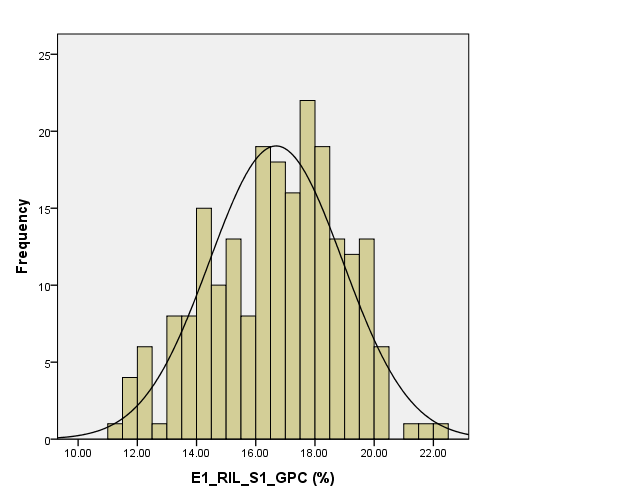

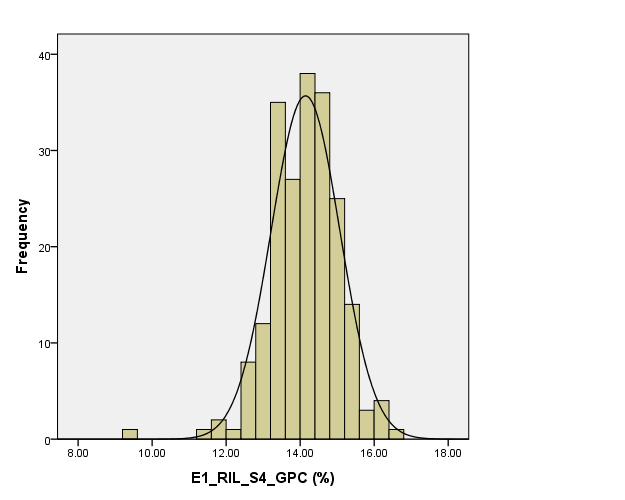

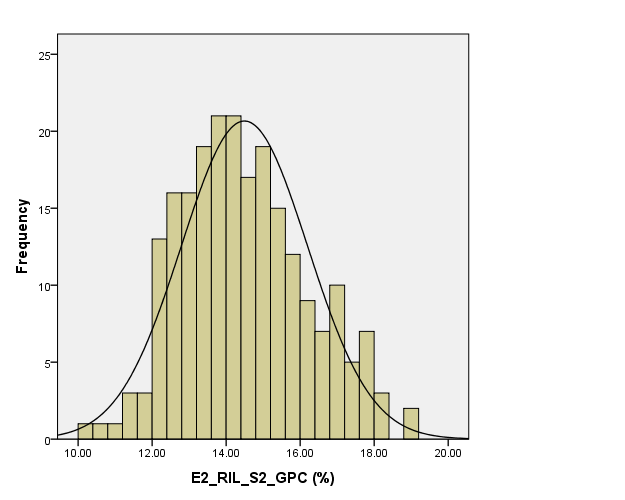

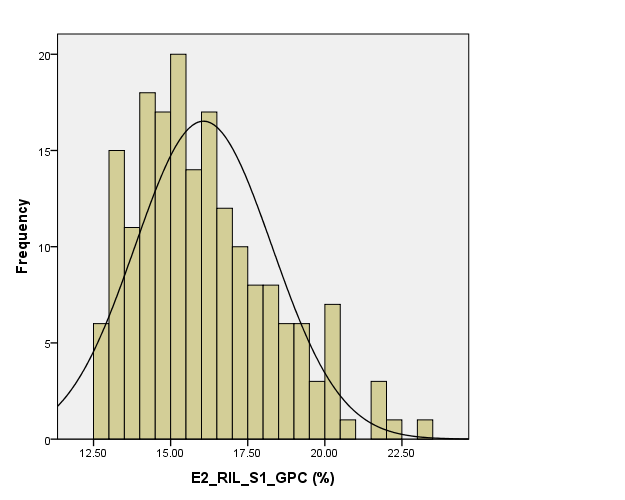

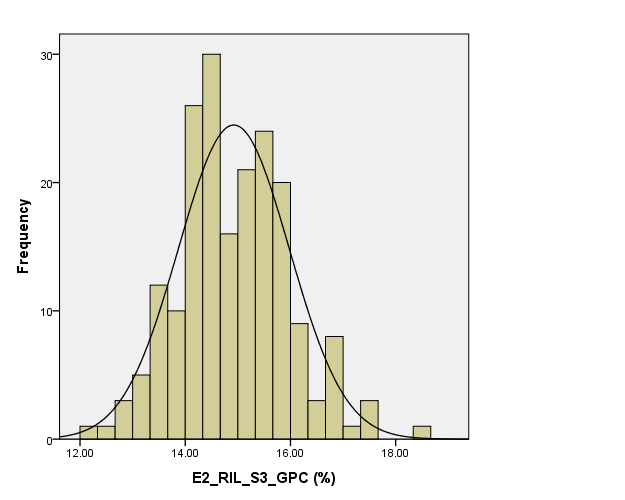

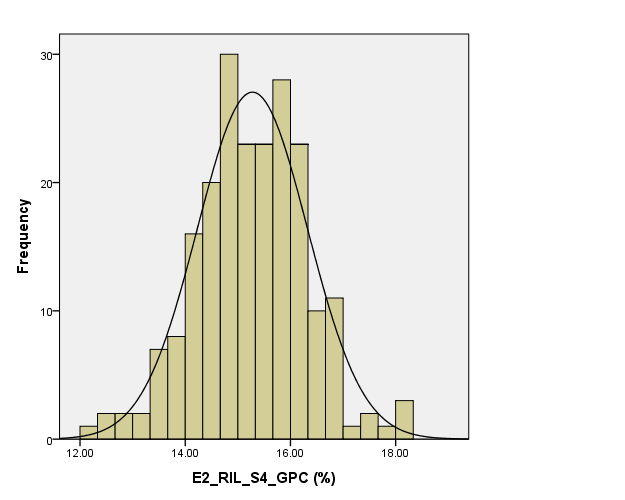


Fig.S1 The frequency bar graphs of grain protein content in four stages in two environments


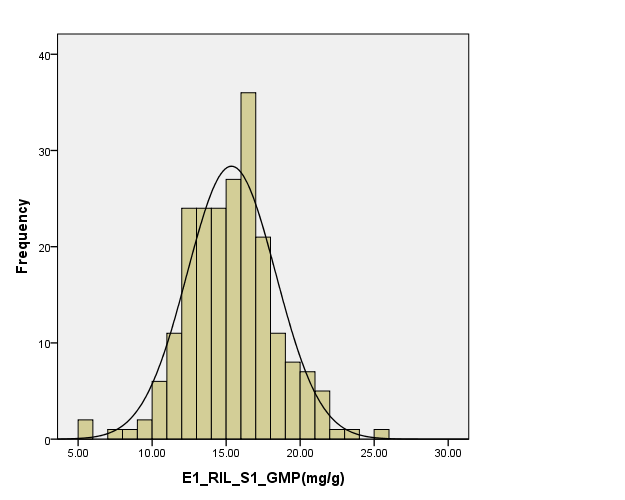

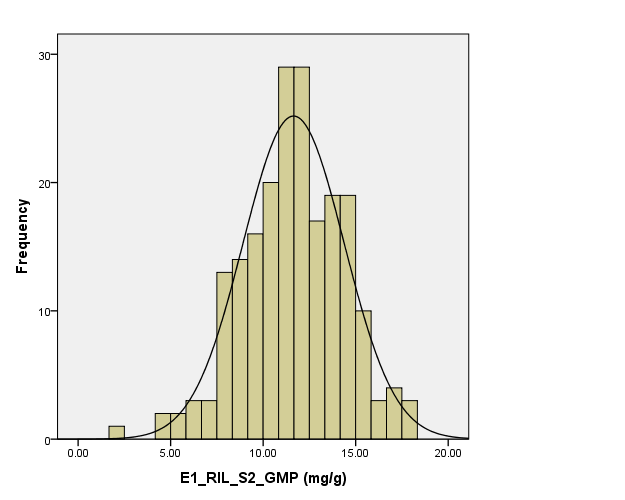

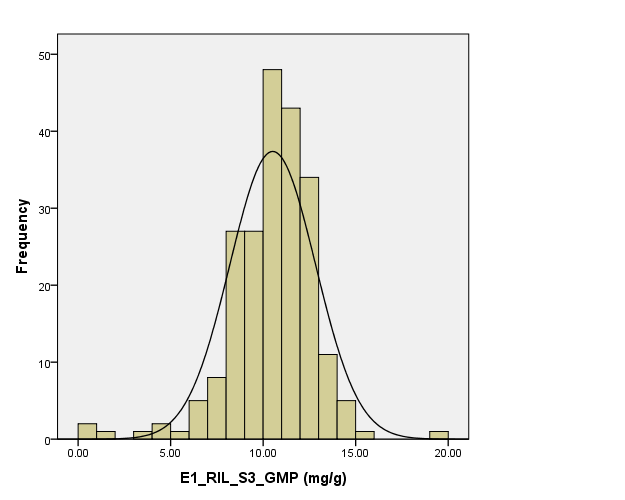

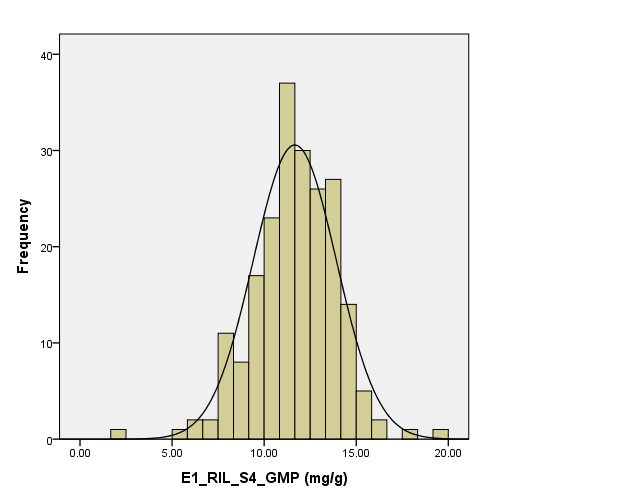

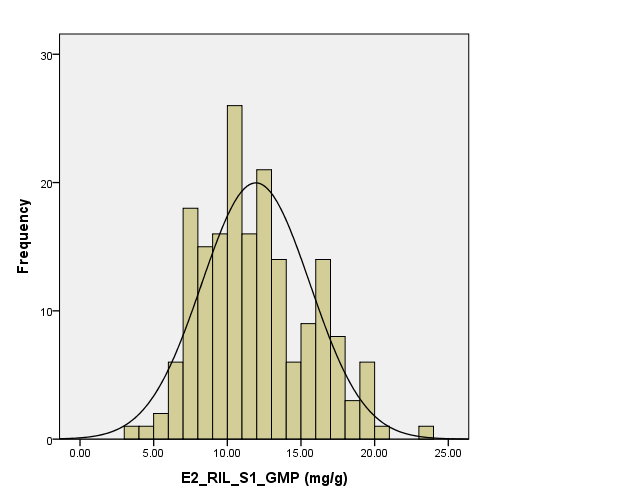

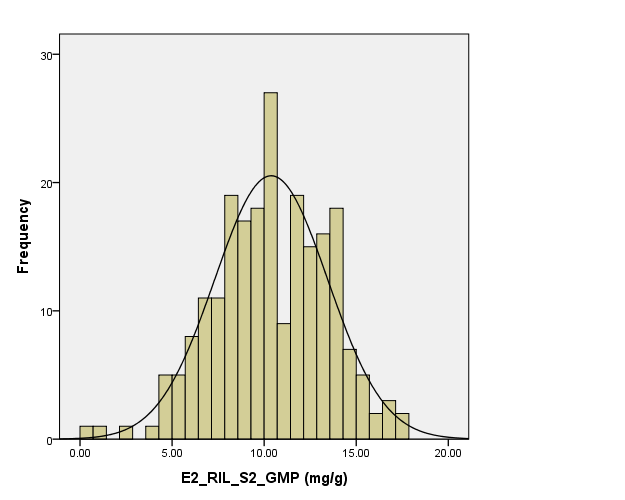

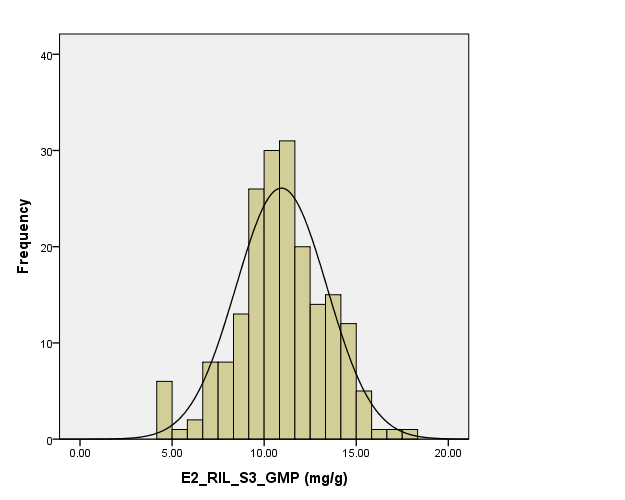

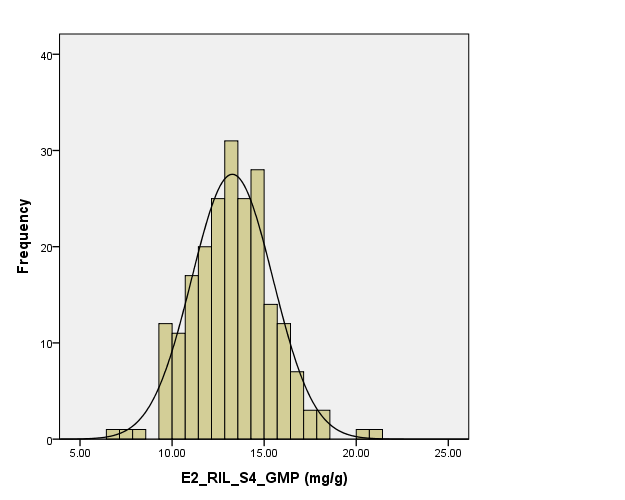


Fig.S2 The frequency bar graphs of glutenin macropolymer content in four stages in two environments


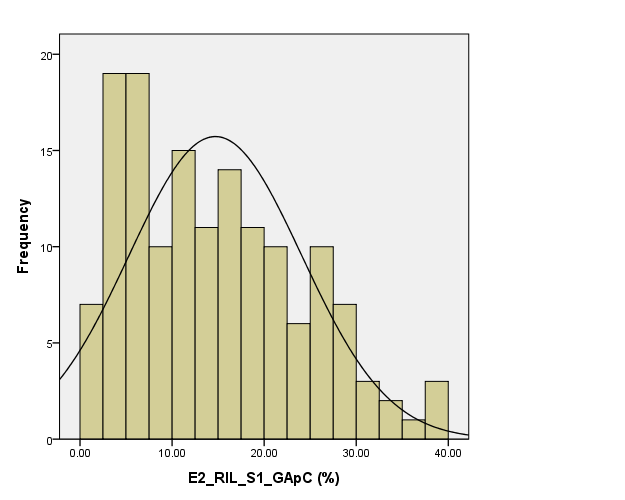

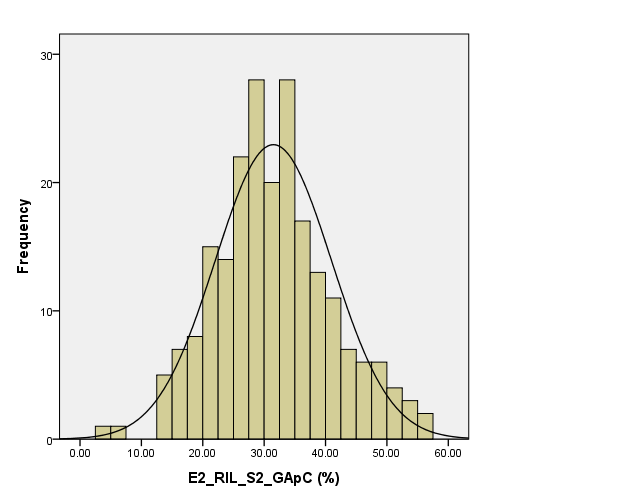

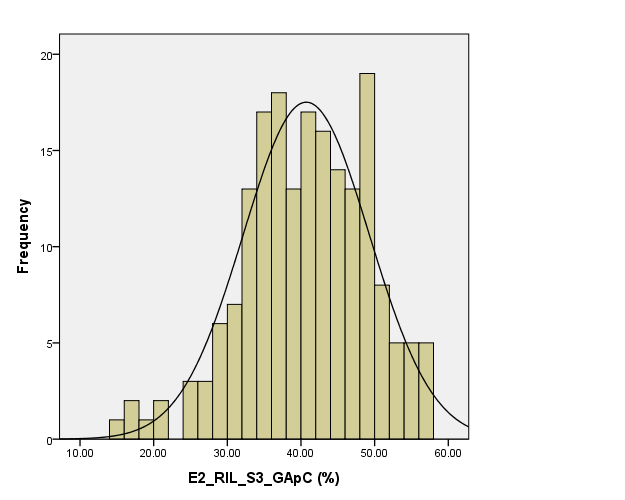

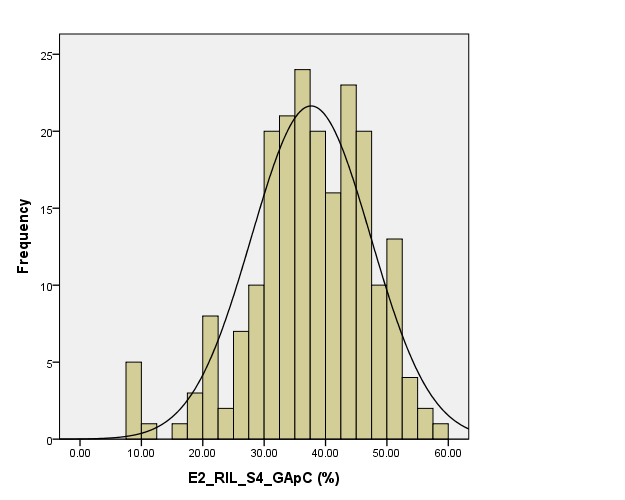

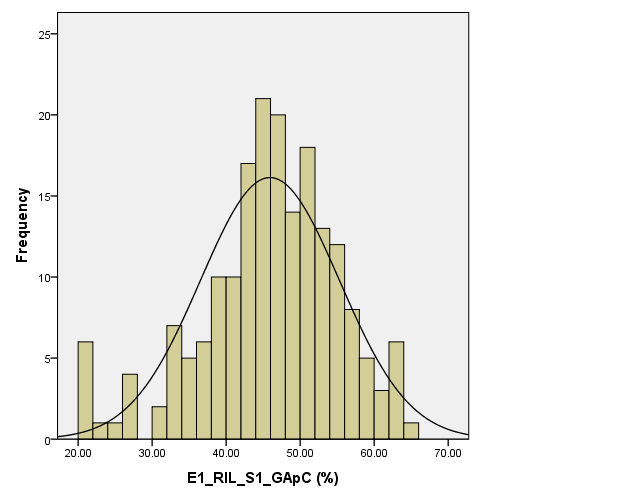

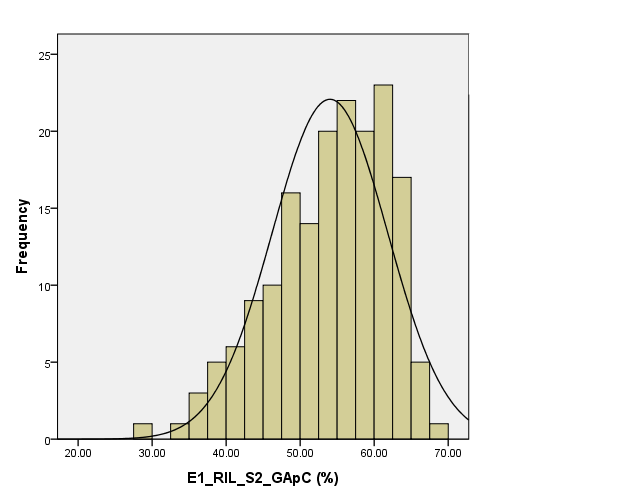

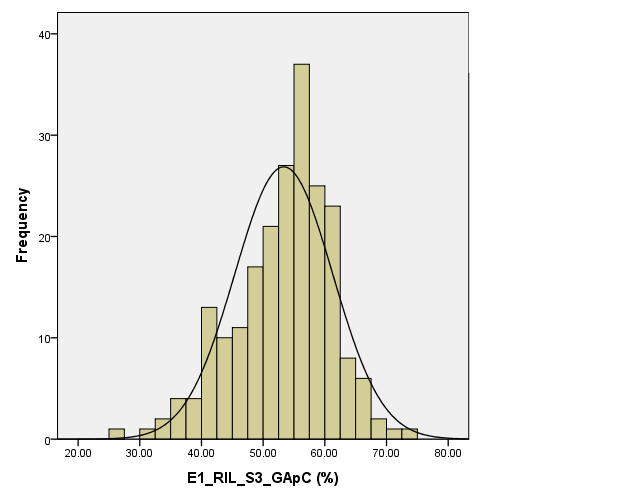

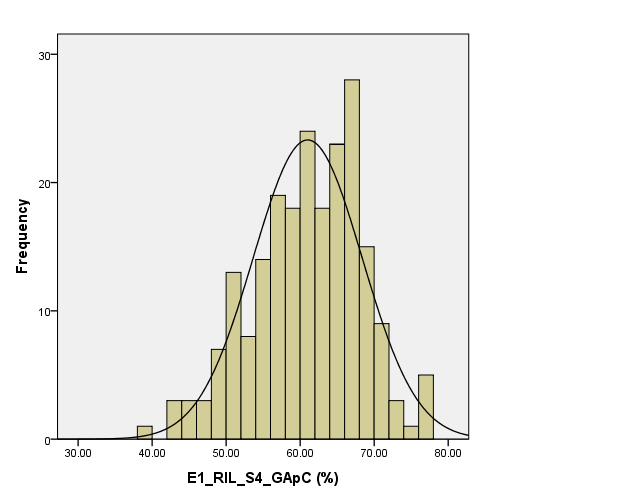


Fig.S3 The frequency bar graphs of amylopectin content in four stages in two environments


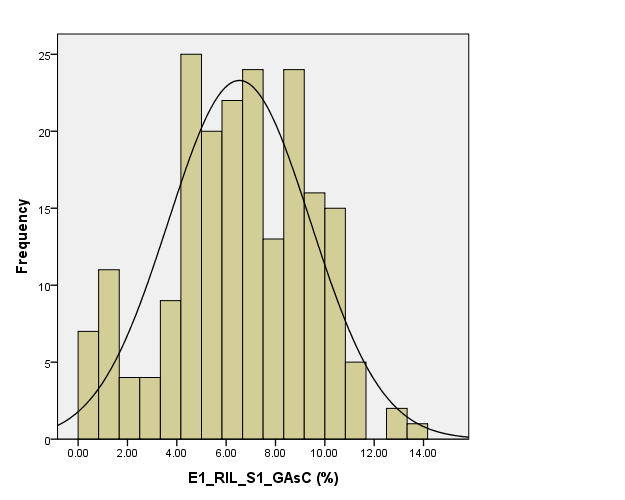

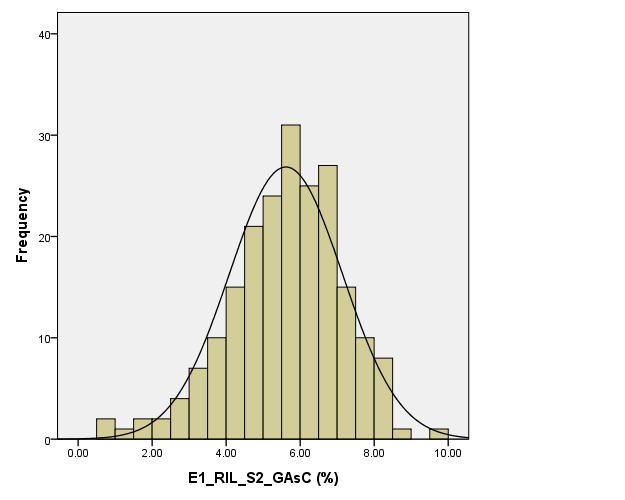

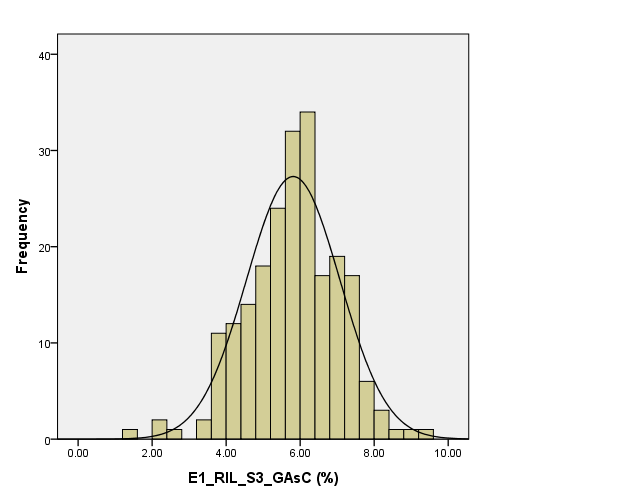

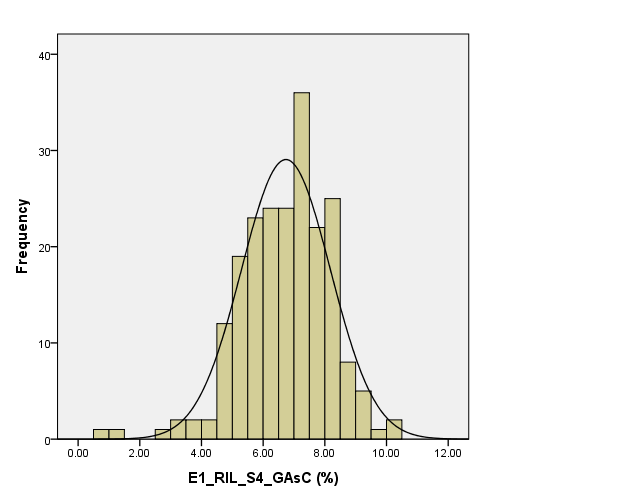

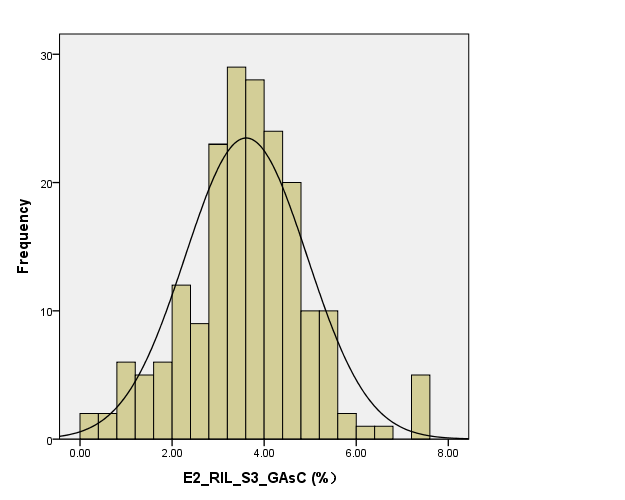

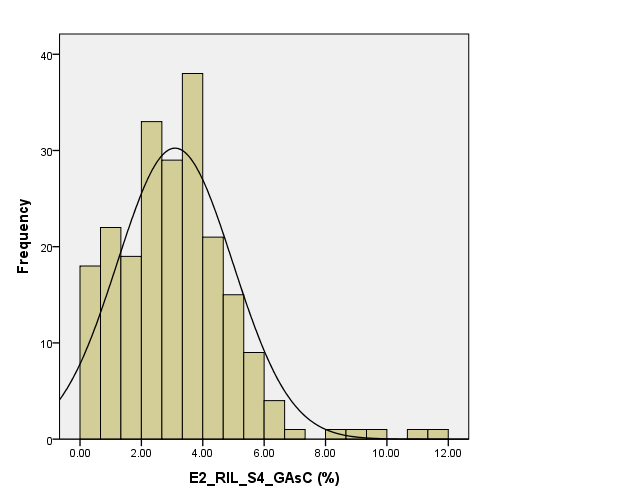


Fig.S4 The frequency bar graphs of amylose content in four stages in two environments


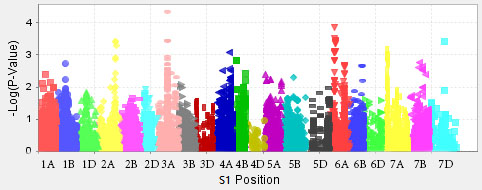

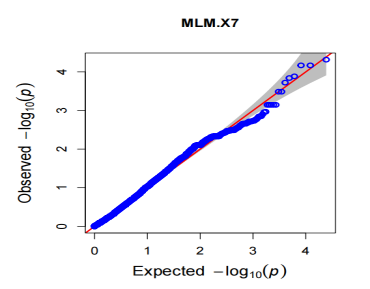

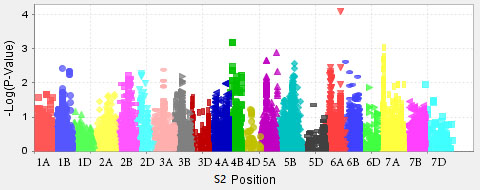

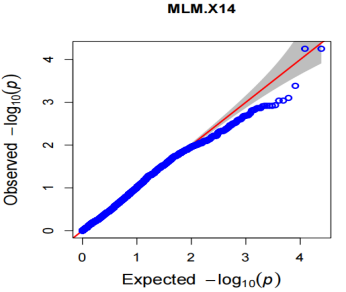

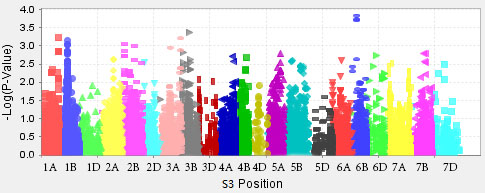

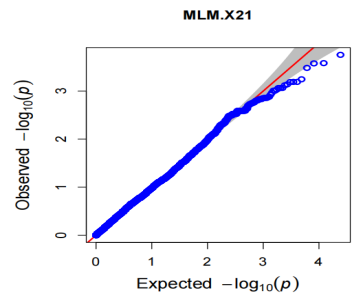

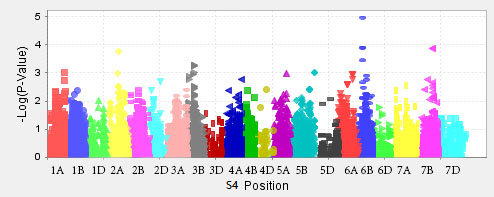

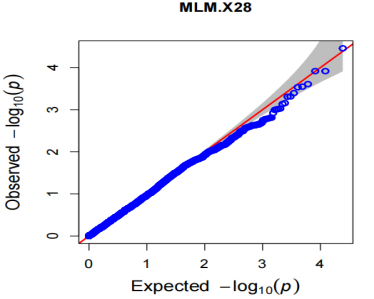


Fig. S5 The Manhattan plot of GPC of wheat nature population by unconditional association analysis in E1


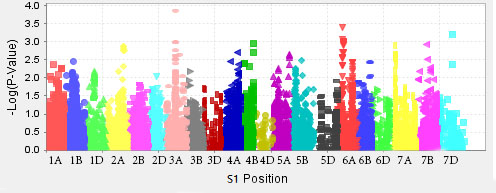

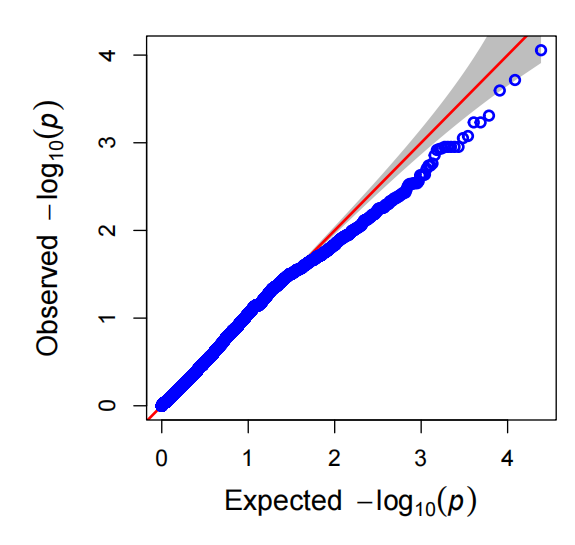

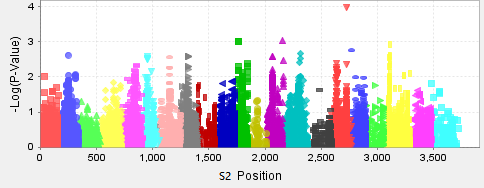

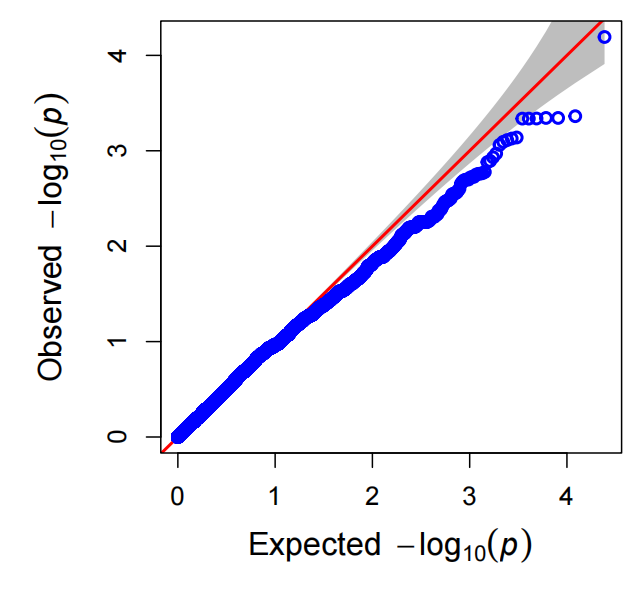

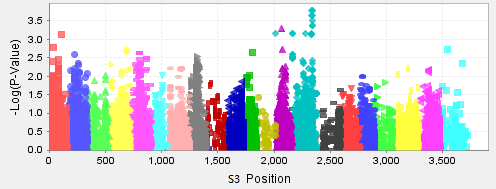

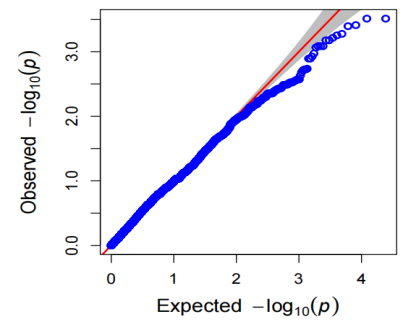

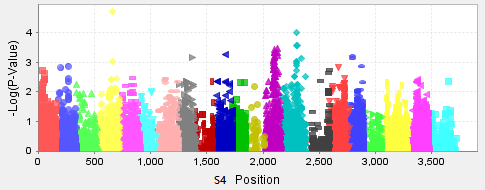

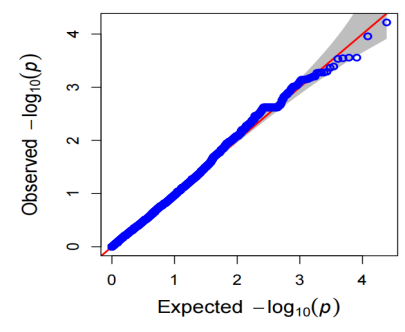


Fig. S6 The Manhattan plot of GMP of wheat nature population by unconditional association analysis in E1


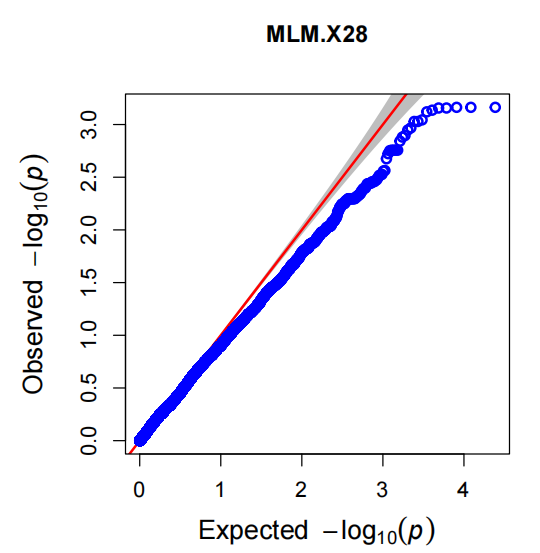

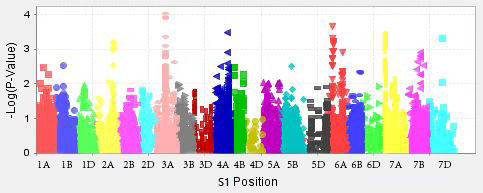

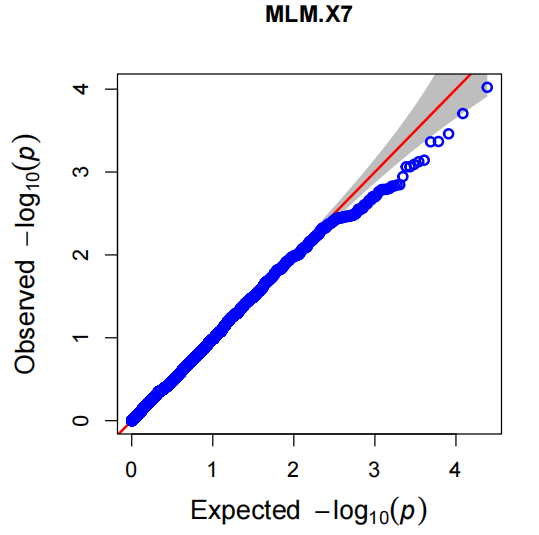

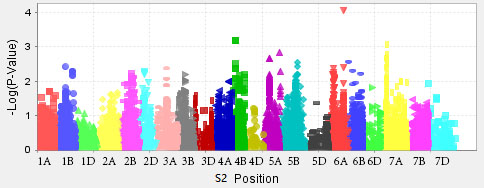

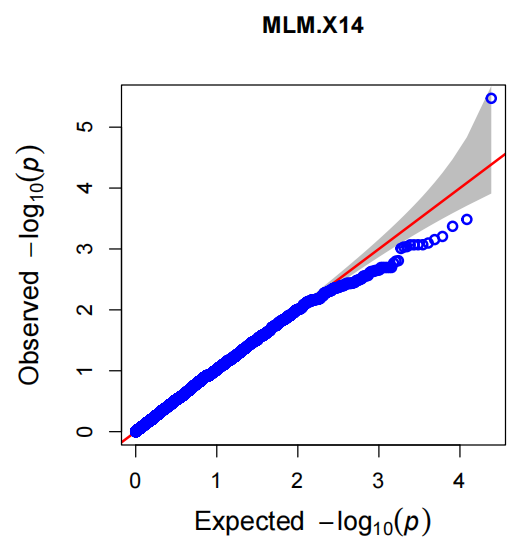

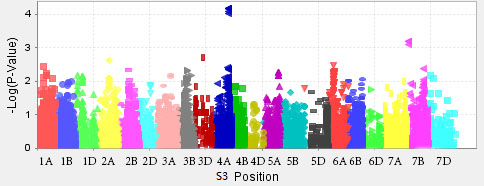

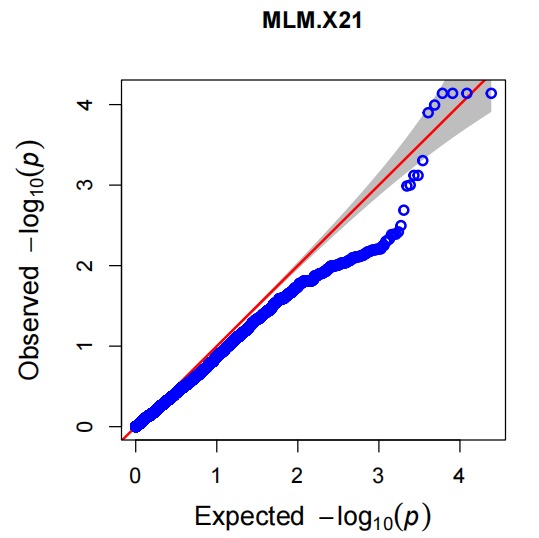

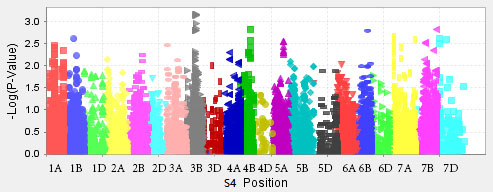


Fig. S7 The Manhattan plot of GApC of wheat nature population by unconditional association analysis in E1


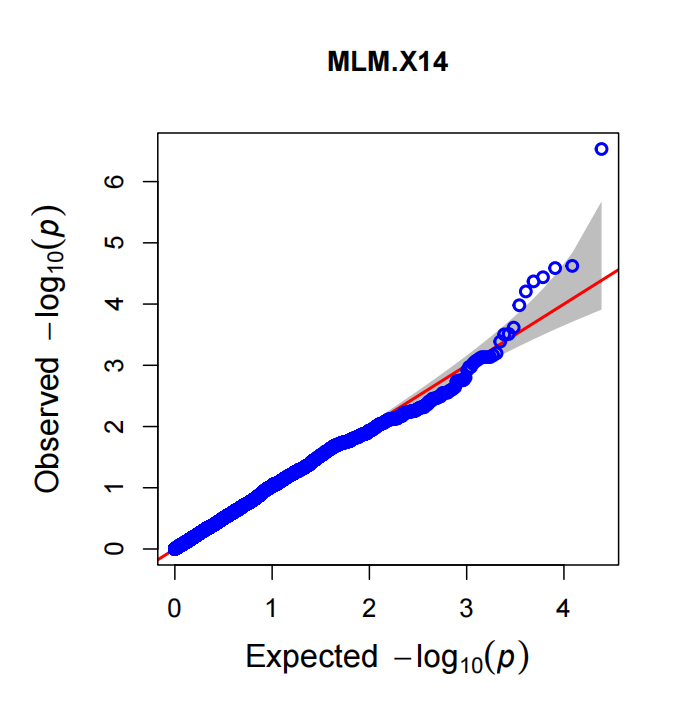

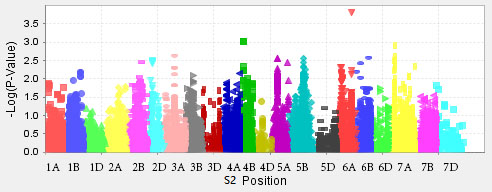

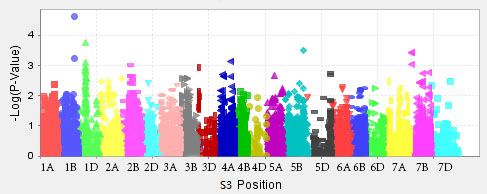

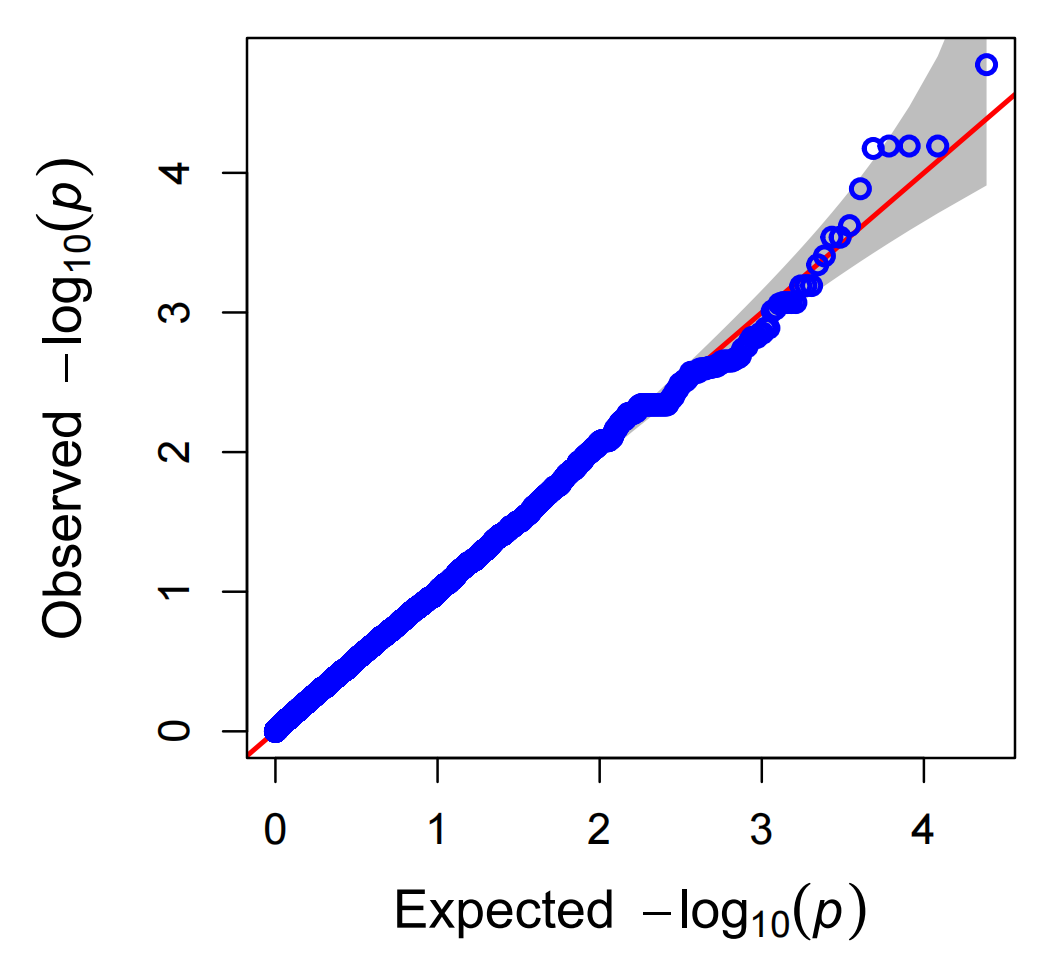

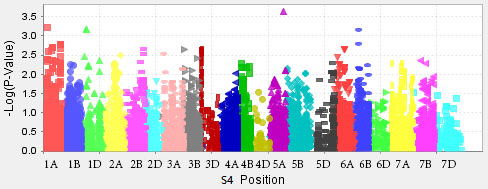

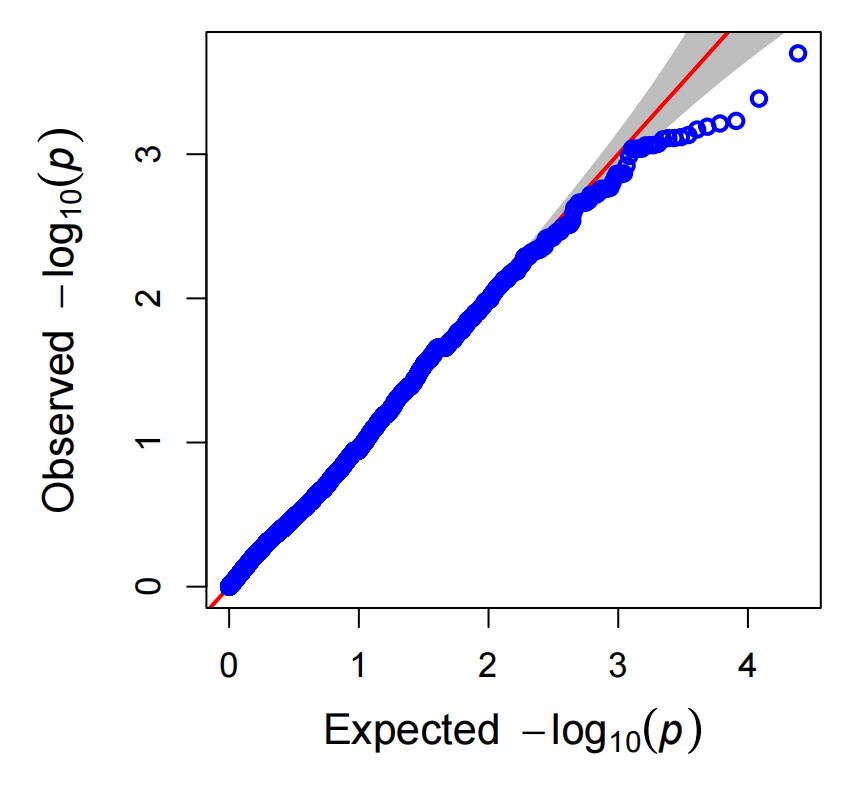

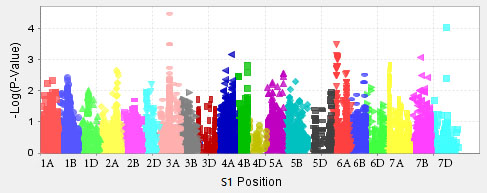

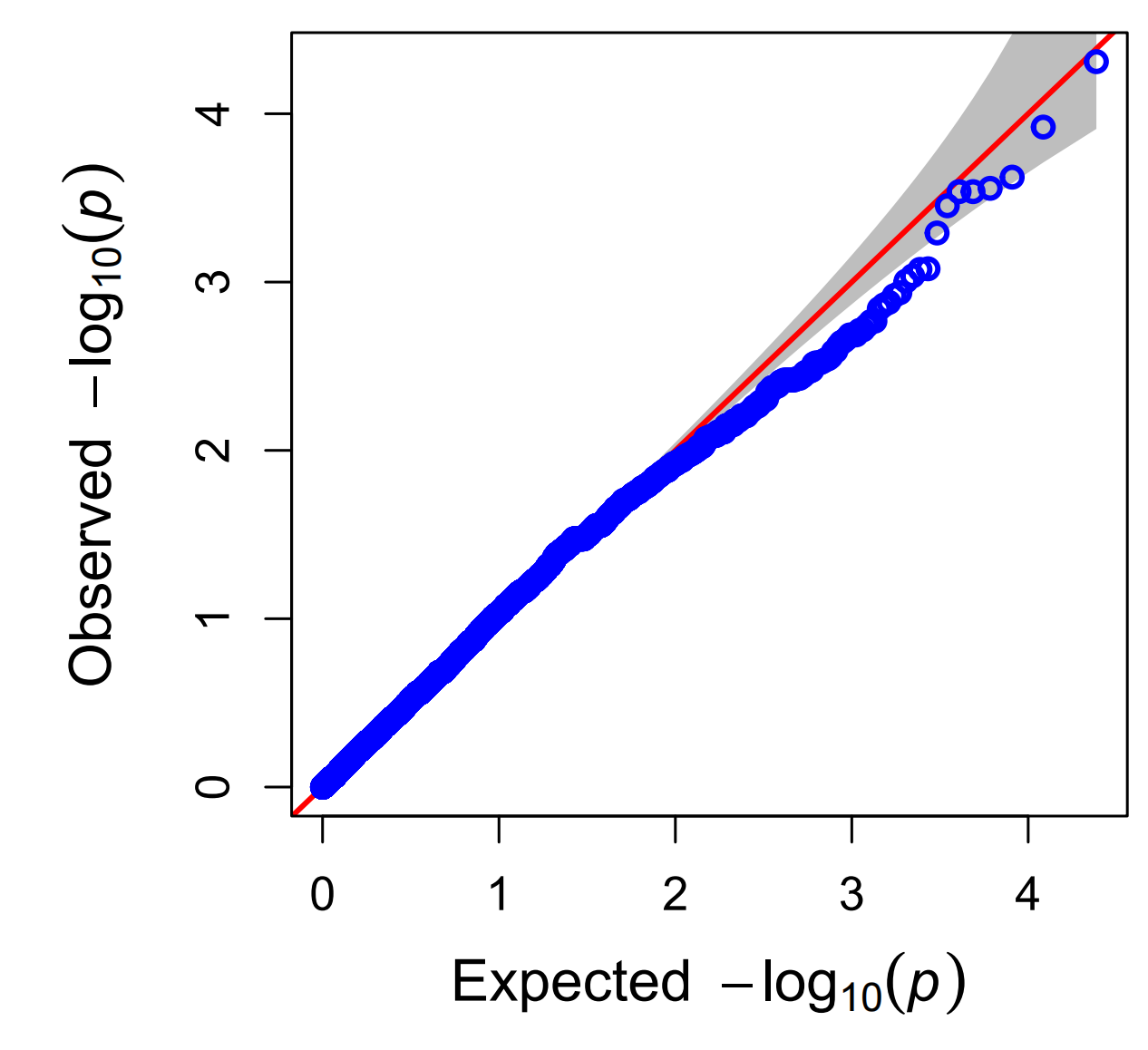


Fig. S8 The Manhattan plot of GAsC of wheat nature population by unconditional association analysis in E1


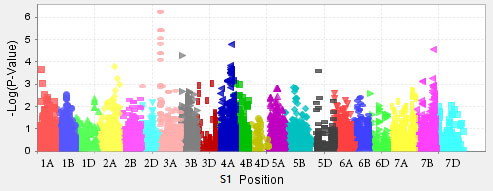

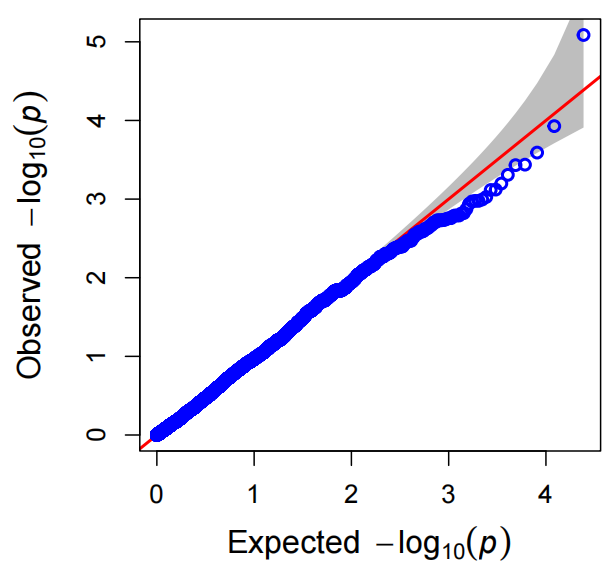

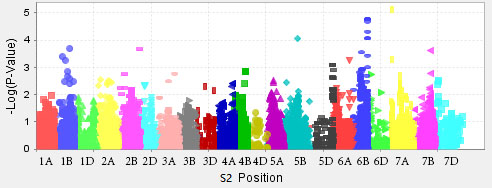

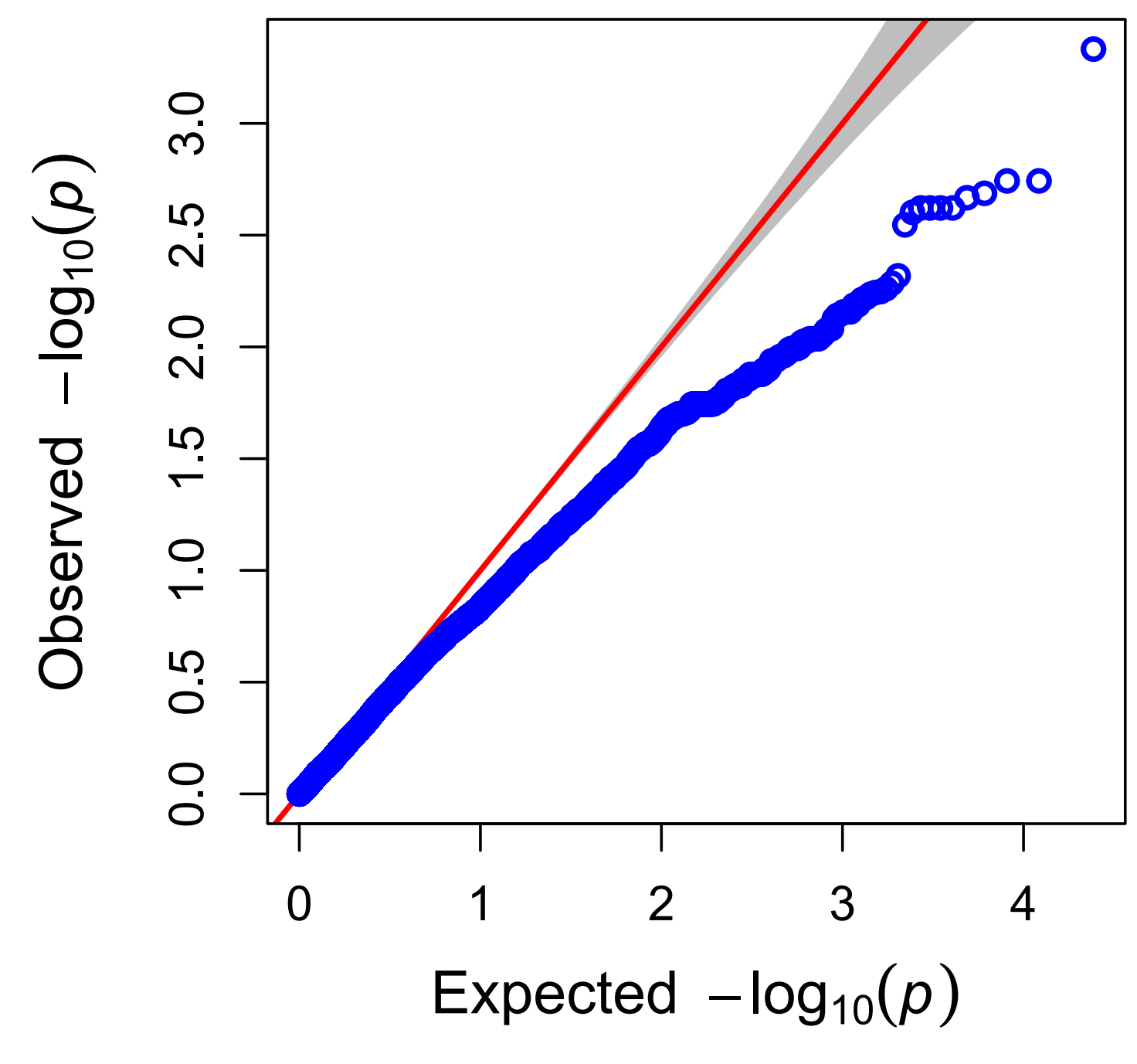

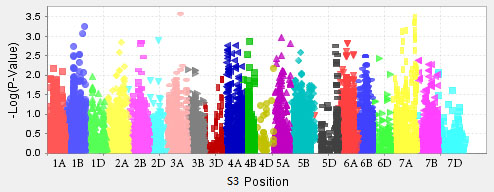

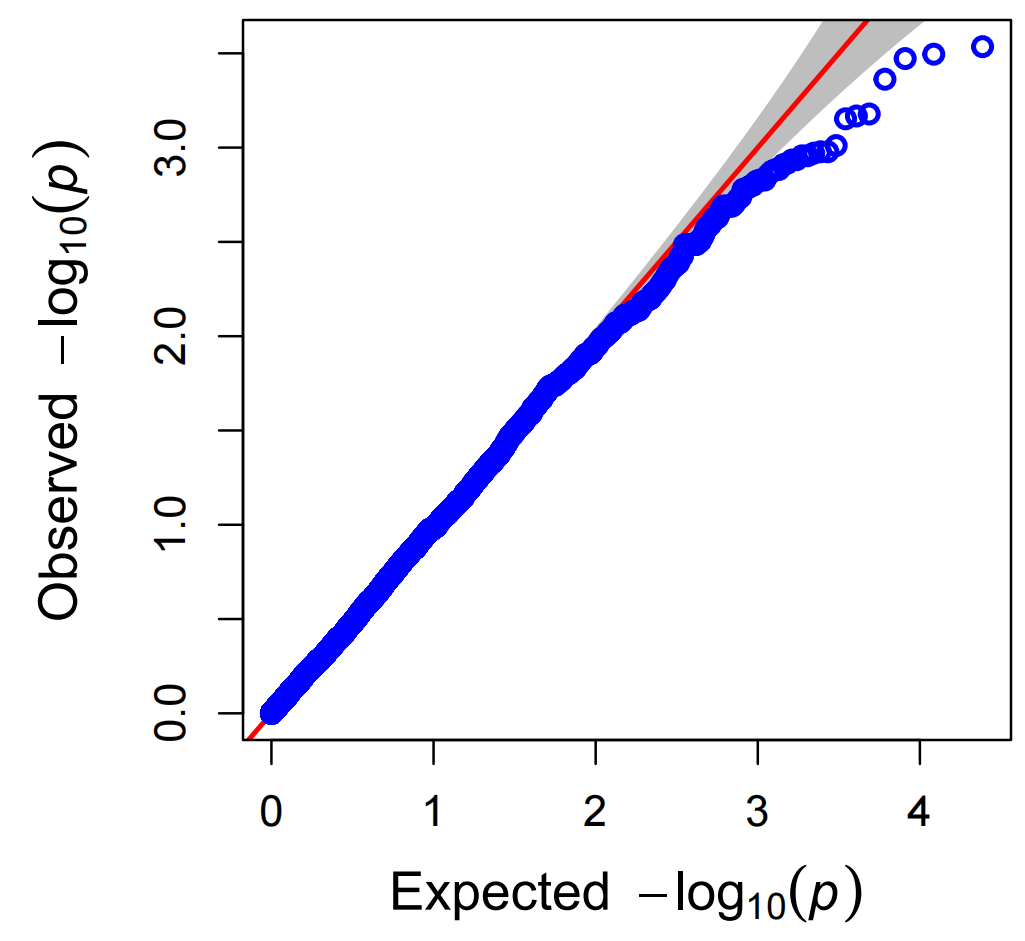

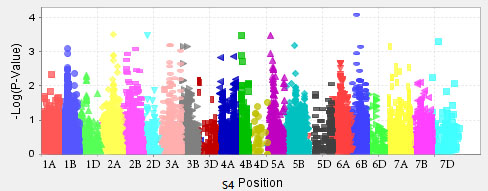

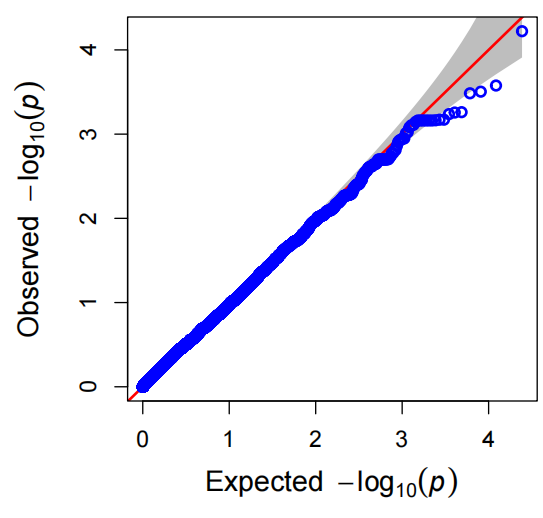


Fig. S9 The Manhattan plot of GPC of wheat nature population by unconditional association analysis in E2


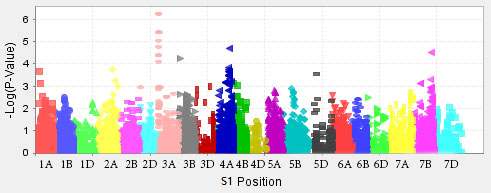

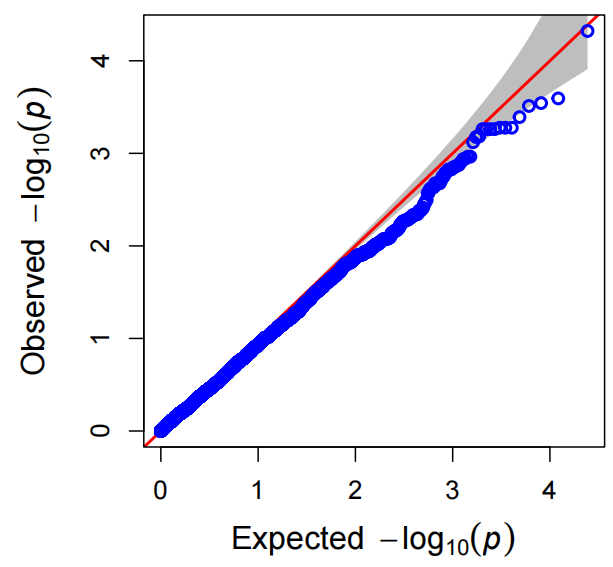

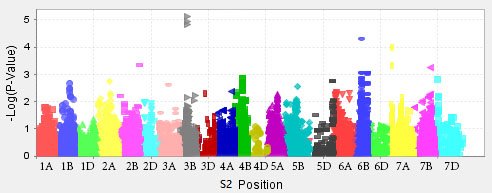

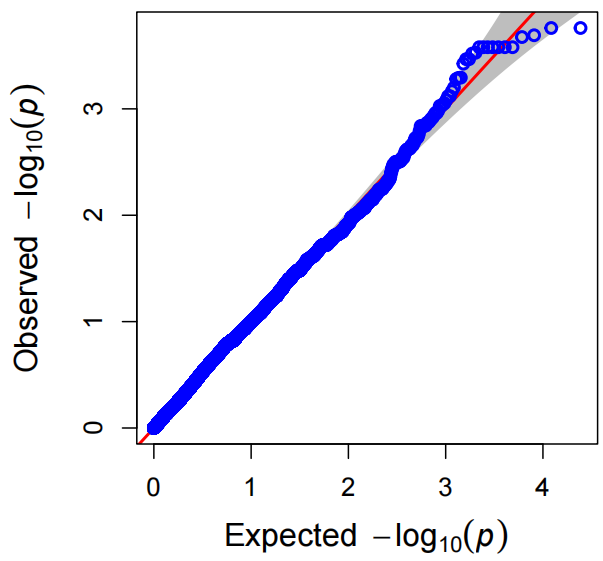

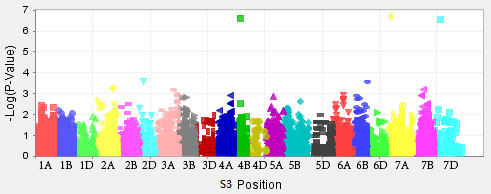

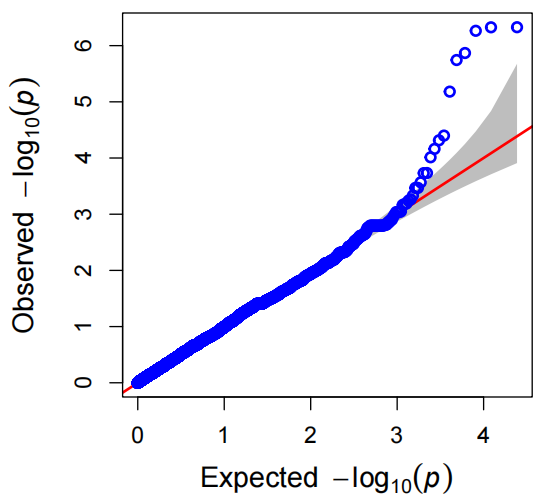

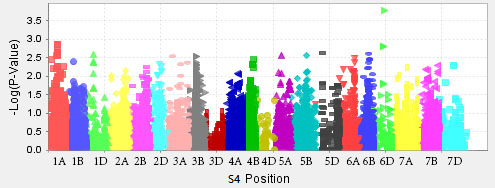

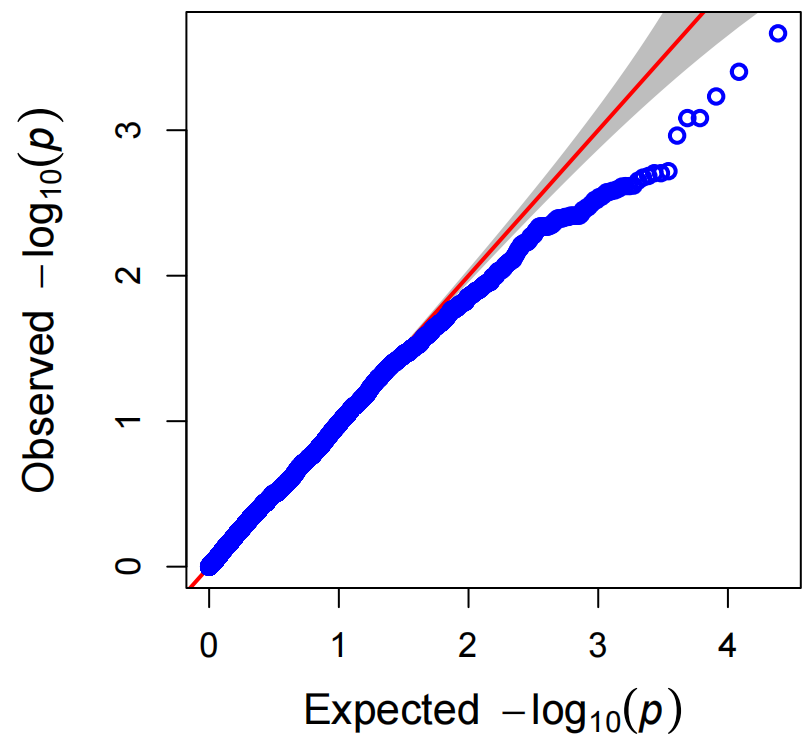


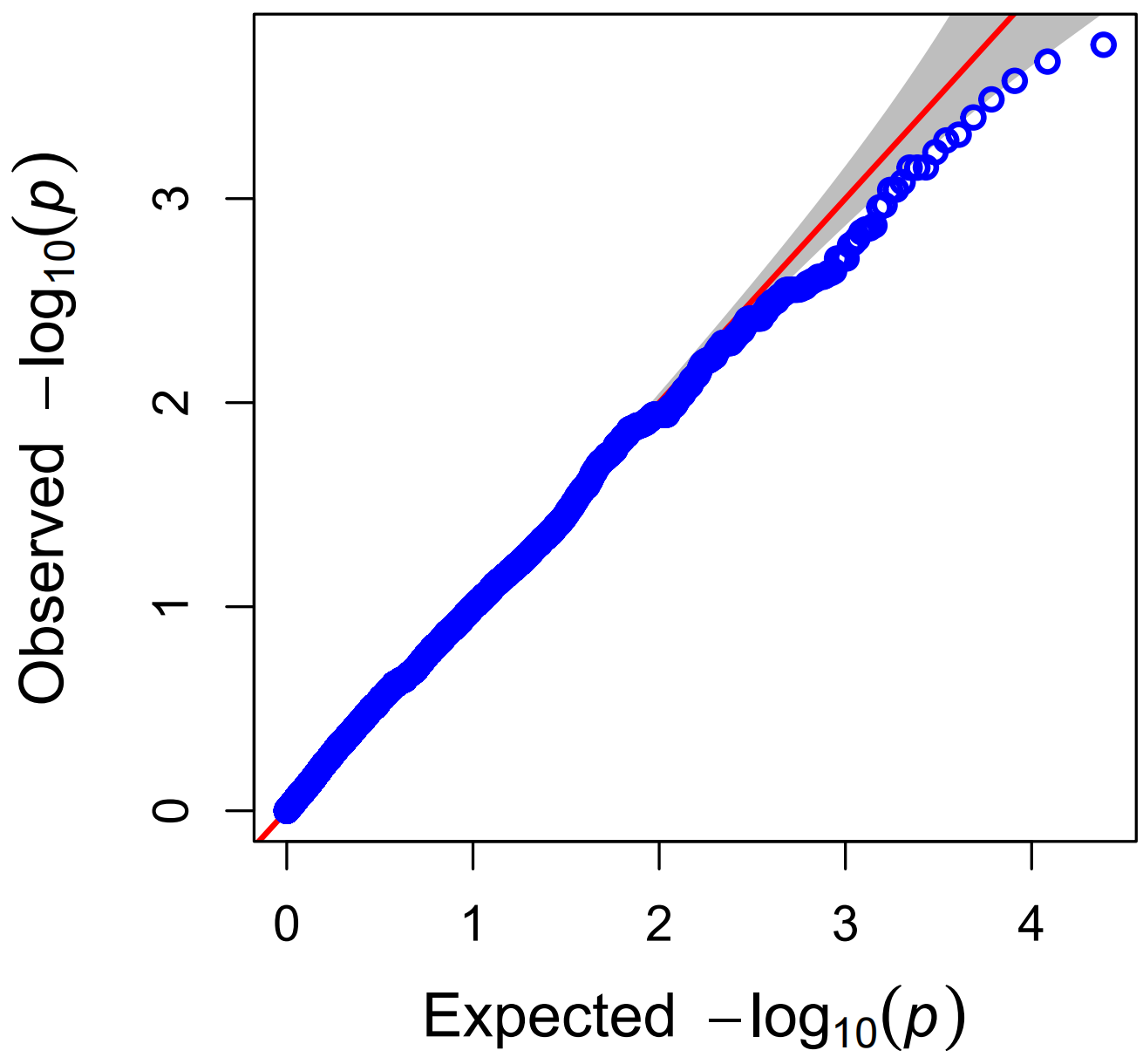

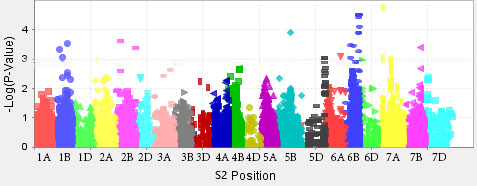
 Fig. S10 The Manhattan plot of GMP of wheat nature population by unconditional association analysis in E2


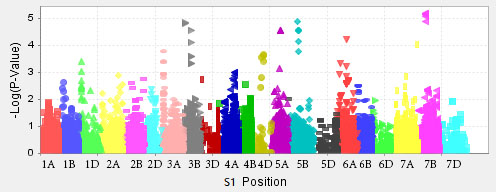

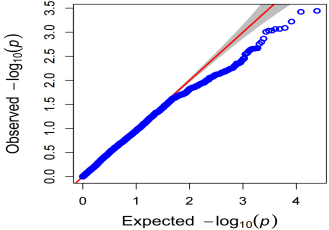

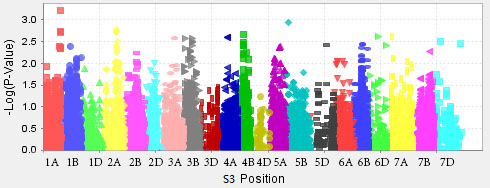

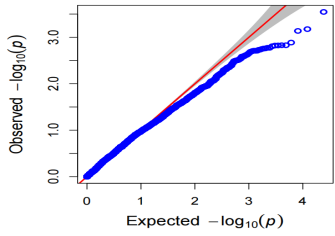

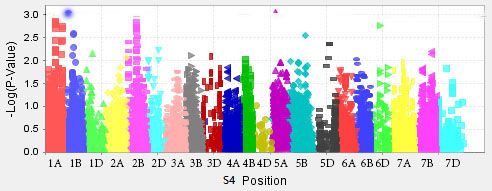

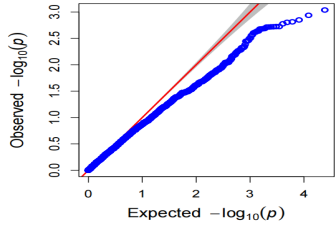

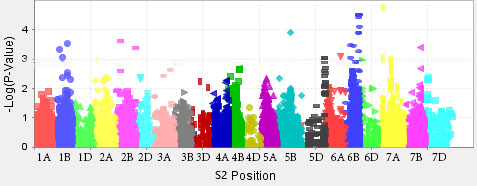

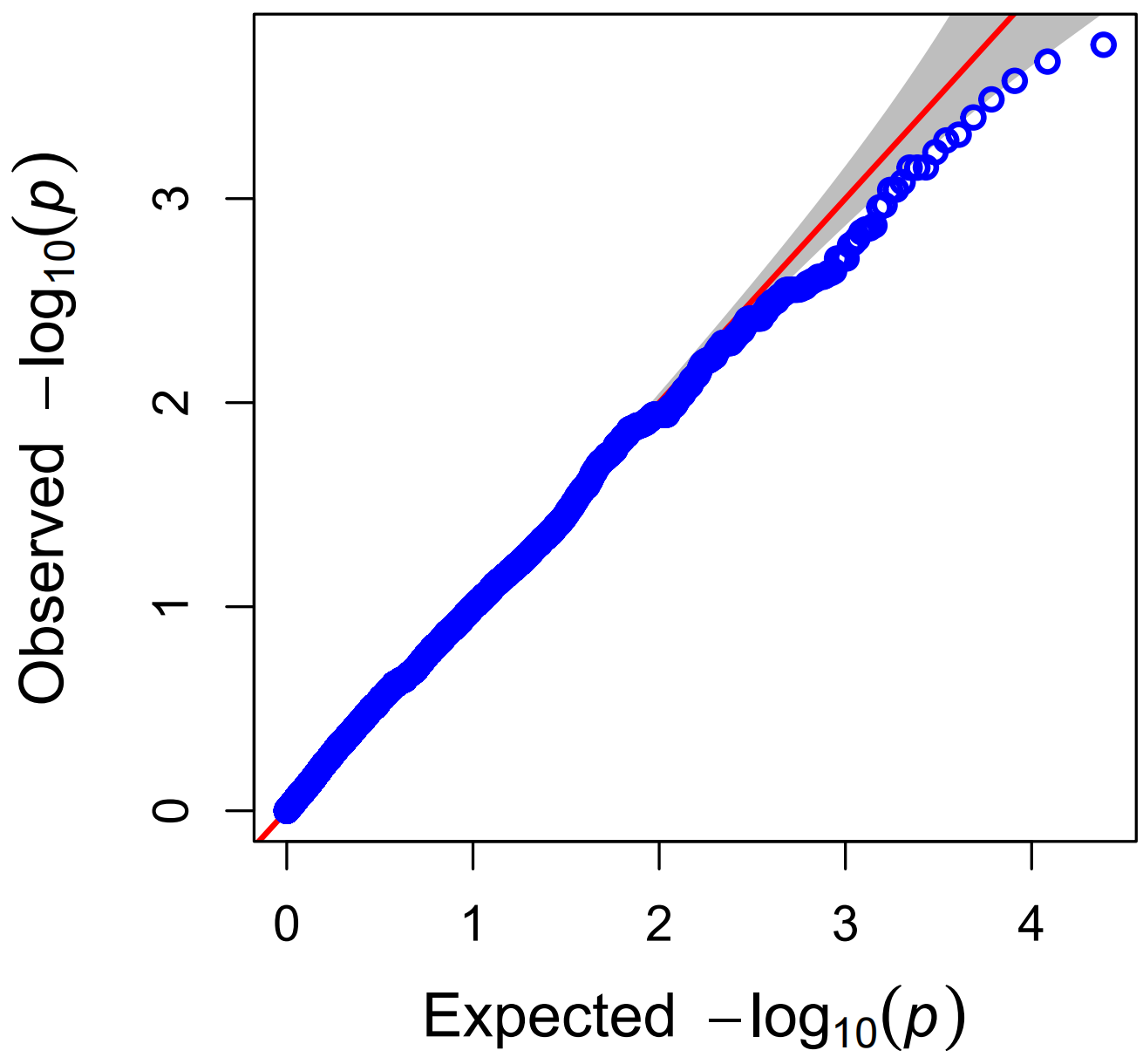


Fig. S11 The Manhattan plot of GApC of wheat nature population by unconditional association analysis in E2


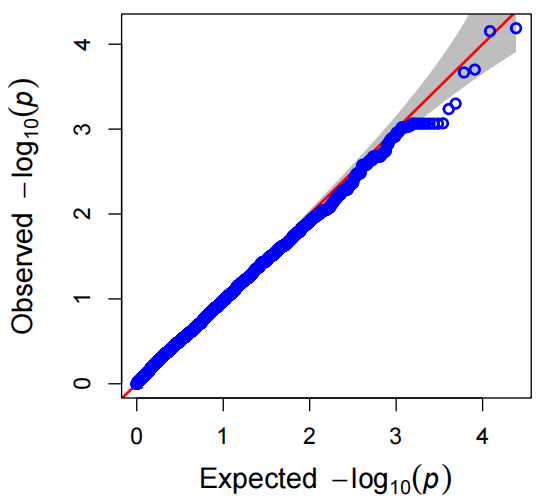

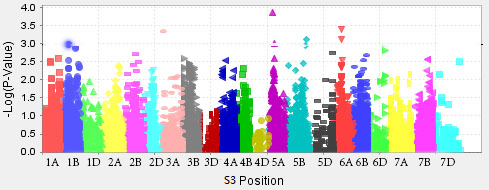

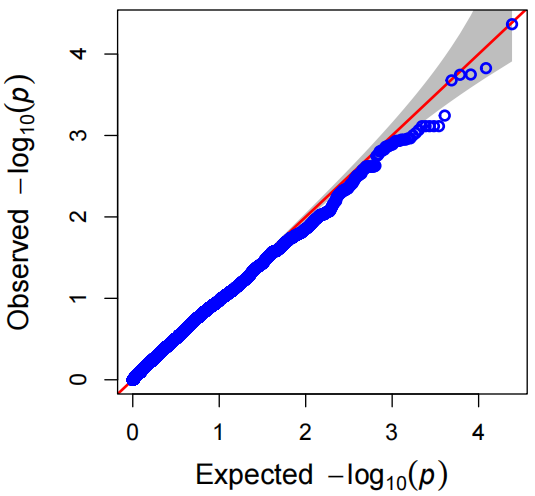

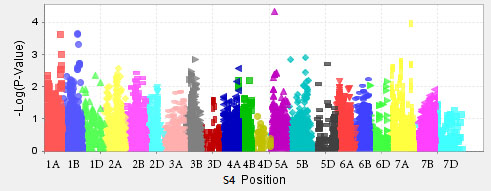


Fig. S12 The Manhattan plot of GAsC of wheat nature population by unconditional association analysis in E2


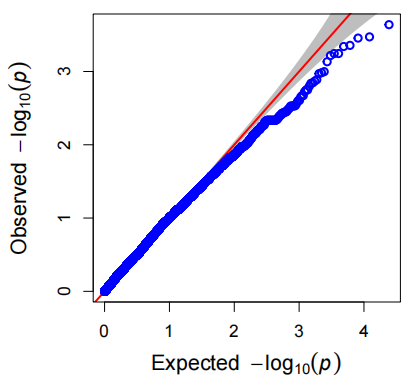

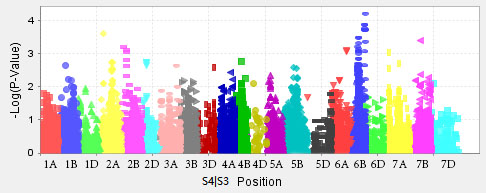

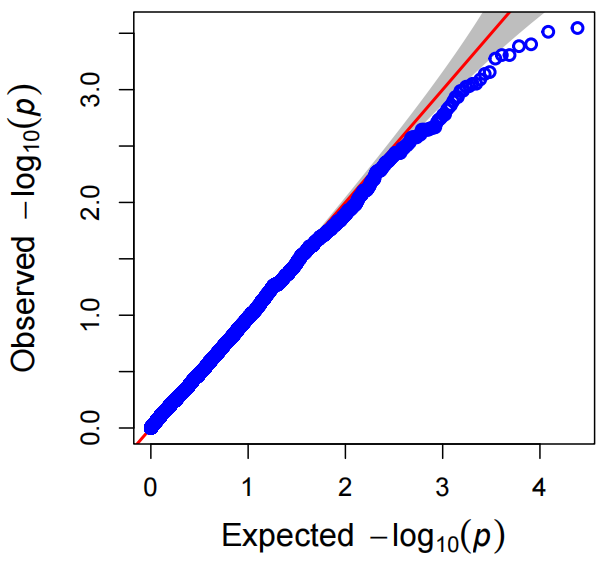

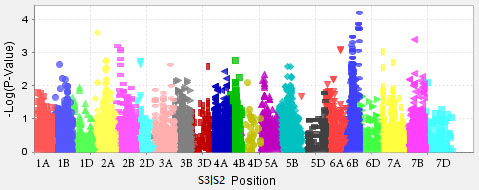

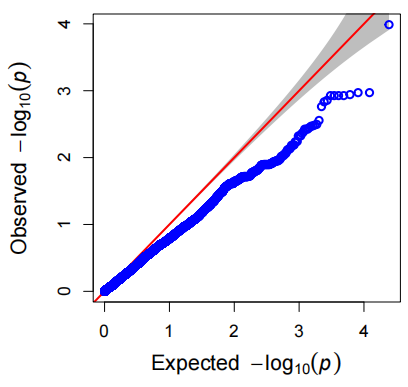

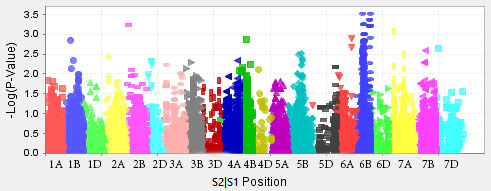


Fig. S13 The Manhattan plot of GPC of wheat nature population by conditional association analysis in E2


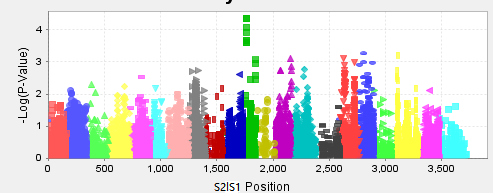

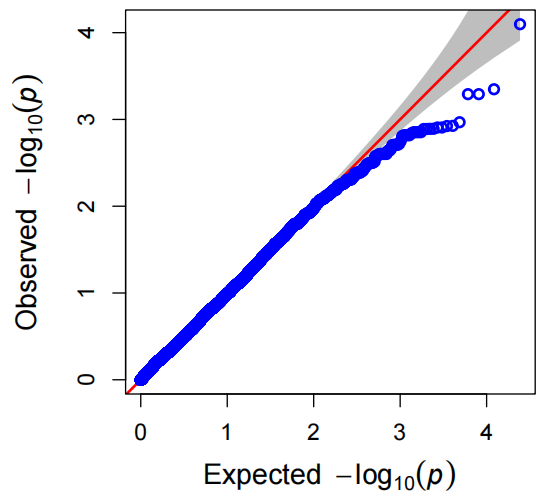


Fig. S14 The Manhattan plot of GApC of wheat nature population by conditional association analysis in E1

Fig. S15 The Manhattan plot of GApC of wheat nature population by conditional association analysis in E2
