# Supplementary material for: Genetic dissection of protein and starch during wheat grain development using QTL mapping and GWAS
Source: Front Plant Sci. 2023 Jun 12;14:1189887. doi: 10.3389/fpls.2023.1189887 (PMC10291175; doi:10.3389/fpls.2023.1189887)

Gene_cluster1

ACCESSION	GENE_NAME
TRAESCS2D02G261300	GATB
TRAESCS2A02G537100	MSD1
TRAESCS3B02G103500	CRK6
TRAESCS3A02G241500	ctpA
TRAESCSU02G123400	AAK6
TRAESCS6B02G206400	NIG
TRAESCS2A02G012600	UGT80A2
TRAESCS7B02G025800	TOPP6
TRAESCS3B02G131500	UBC2
TRAESCS5D02G124500	PYRB
TRAESCS6A02G203600	BAK1
TRAESCS3A02G261200	FYPP
TRAESCS3A02G342500	UBC8
TRAESCS3B02G334400	SERK4
TRAESCS6D02G171400	PP2A1
TRAESCS3B02G341000	UBP26
TRAESCS2A02G238400	ZDS1
TRAESCS3B02G401200	HUB1
TRAESCS3A02G241500	ctpA
TRAESCS3B02G070700	COAC2
TRAESCS4A02G214100	TOL3
TRAESCS7D02G344000	SBE1

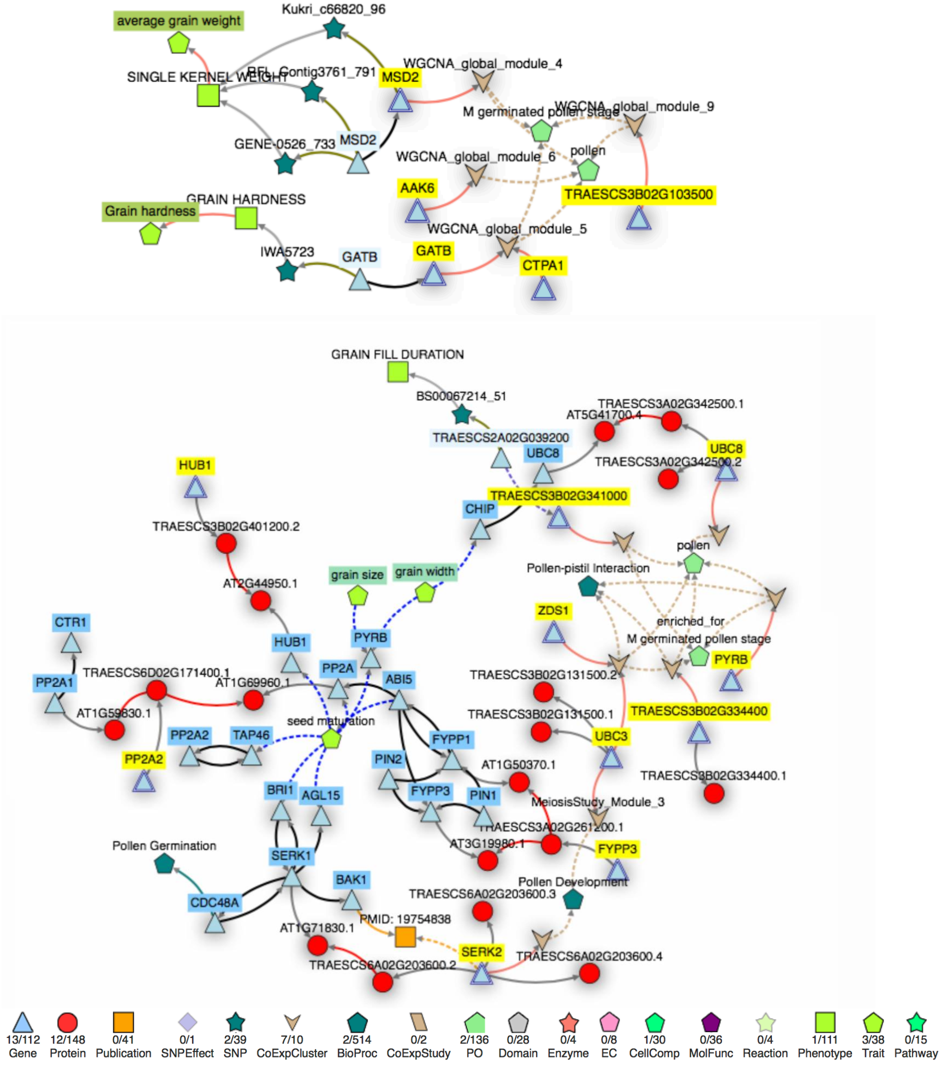

Gene_cluster2

ACCESSION	GENE_NAME
TRAESCS7D02G117800	SS1
TRAESCS3B02G211500	EAAC
TRAESCS2B02G267200	
TRAESCS3B02G246600	KIN8A
TRAESCS2D02G308600	SBE1
TRAESCS3A02G207800	SEC61B
TRAESCS7B02G305500	
TRAESCS3A02G305900	CIPK12

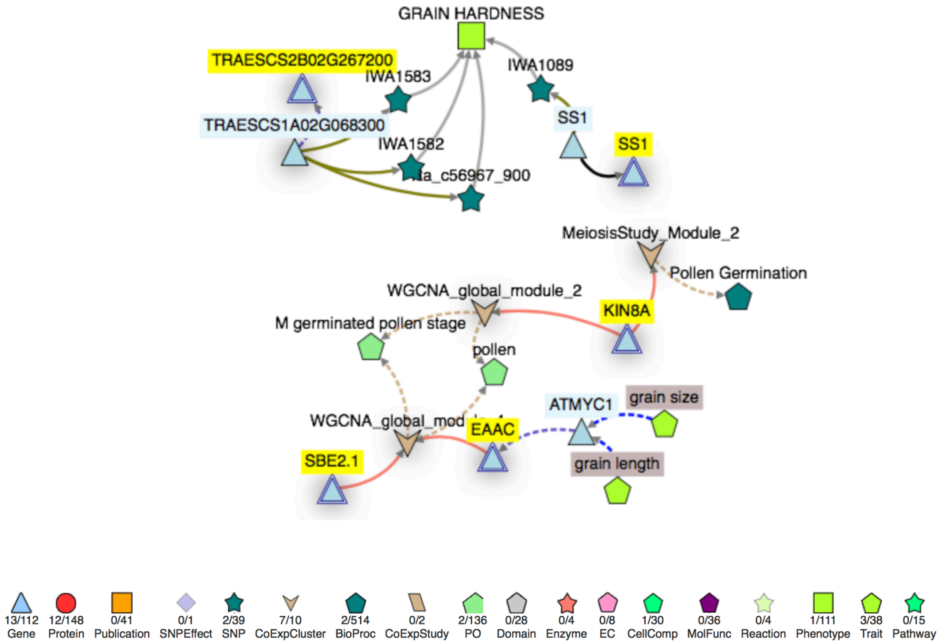

Gene_cluster3

ACCESSION	GENE_NAME
TRAESCS7D02G064300	WAXY
TRAESCS1B02G368500	SS3
TRAESCS5D02G255800	ISA3
TRAESCS3A02G112600	UBC2
TRAESCS6A02G298100	GLN1-1
TRAESCS6A02G139400	CRK6
TRAESCS7B02G069800	ER1
TRAESCS3B02G403700	

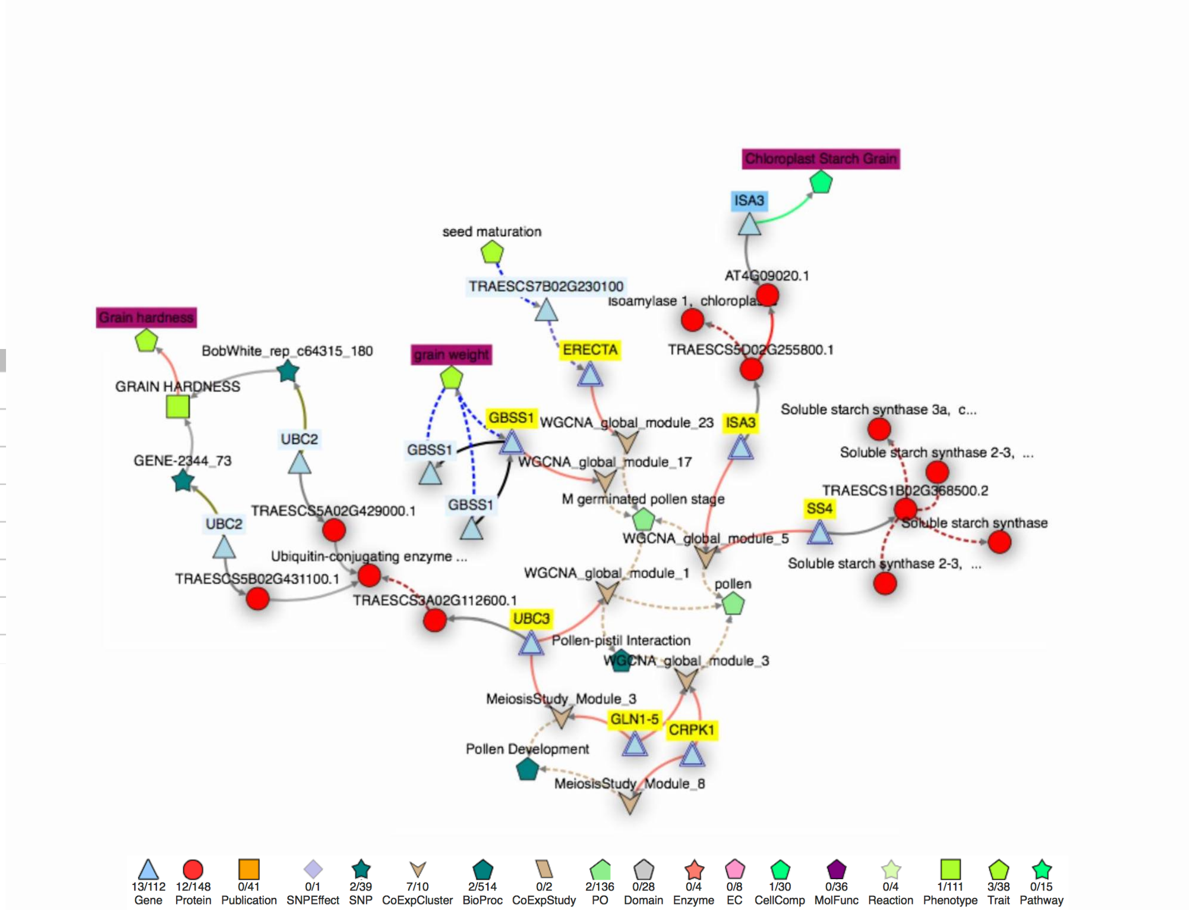

Gene_cluster4

ACCESSION	GENE_NAME
TRAESCS7A02G070100	WAXY
TRAESCS5A02G326100	CCP1
TRAESCS5D02G059700	NAC077
TRAESCS3B02G021900	XYXT1
TRAESCS5B02G054200	NAC077

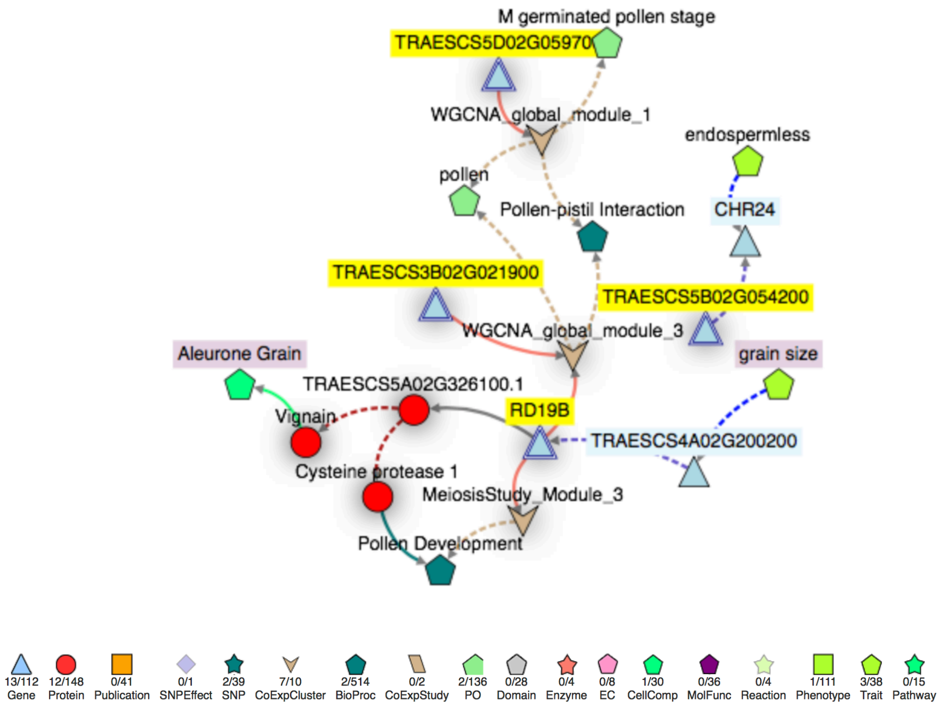

Supplement: Supplementary file 5 [file DataSheet_1.pdf]
